# Moderate Fields, Maximum Potential: Achieving High Records with Temperature-Stable Energy Storage in Lead-Free BNT-Based Ceramics

**DOI:** 10.1007/s40820-023-01290-4

**Published:** 2024-01-18

**Authors:** Wenjing Shi, Leiyang Zhang, Ruiyi Jing, Yunyao Huang, Fukang Chen, Vladimir Shur, Xiaoyong Wei, Gang Liu, Hongliang Du, Li Jin

**Affiliations:** 1 Electronic Materials Research Laboratory, Key Laboratory of the Ministry of Education, School of Electronic Science and Engineering, Xi’an Jiaotong University, Xi’an, 710049 People’s Republic of China; 2 School of Materials and Energy, Southwest University, Chongqing, 400715 People’s Republic of China; 3 School of Natural Sciences and Mathematics, Ural Federal University, Ekaterinburg, 620000 Russia; 4 Multifunctional Electronic Ceramics Laboratory, College of Engineering, Xi’an International University, Xi’an, 710077 People’s Republic of China

**Keywords:** BNT, Energy storage, Lead-free, Relaxor ferroelectrics, Capacitors

## Abstract

The increasing awareness of environmental concerns has prompted a surge in the exploration of lead-free, high-power ceramic capacitors. Ongoing efforts to develop lead-free dielectric ceramics with exceptional energy-storage performance (ESP) have predominantly relied on multi-component composite strategies, often accomplished under ultrahigh electric fields. However, this approach poses challenges in insulation and system downsizing due to the necessary working voltage under such conditions. Despite extensive study, bulk ceramics of (Bi_0.5_Na_0.5_)TiO_3_ (BNT), a prominent lead-free dielectric ceramic family, have seldom achieved a recoverable energy-storage (ES) density (*W*_rec_) exceeding 7 J cm^−3^. This study introduces a novel approach to attain ceramic capacitors with high ESP under moderate electric fields by regulating permittivity based on a linear dielectric model, enhancing insulation quality, and engineering domain structures through chemical formula optimization. The incorporation of SrTiO_3_ (ST) into the BNT matrix is revealed to reduce the dielectric constant, while the addition of Bi(Mg_2/3_Nb_1/3_)O_3_ (BMN) aids in maintaining polarization. Additionally, the study elucidates the methodology to achieve high ESP at moderate electric fields ranging from 300 to 500 kV cm^−1^. In our optimized composition, 0.5(Bi_0.5_Na_0.4_K_0.1_)TiO_3_–0.5(2/3ST-1/3BMN) (B-0.5SB) ceramics, we achieved a *W*_rec_ of 7.19 J cm^−3^ with an efficiency of 93.8% at 460 kV cm^−1^. Impressively, the B-0.5SB ceramics exhibit remarkable thermal stability between 30 and 140 °C under 365 kV cm^−1^, maintaining a *W*_rec_ exceeding 5 J cm^−3^. This study not only establishes the B-0.5SB ceramics as promising candidates for ES materials but also demonstrates the feasibility of optimizing ESP by modifying the dielectric constant under specific electric field conditions. Simultaneously, it provides valuable insights for the future design of ceramic capacitors with high ESP under constraints of limited electric field.

**Supplementary Information:**

The online version contains supplementary material available at 10.1007/s40820-023-01290-4.

## Highlights

- Achieving ultrahigh energy-storage density (7.19 J cm^−3^) and outstanding storage efficiency (93.8%) at 460 kV cm^−1^ in BNT-based relaxor ferroelectric ceramics under a moderate electric field.
- Superior energy-storage performance accomplished through meticulous regulation of permittivity, enhancement of insulation quality, and strategic domain engineering via chemical formula optimization.
- The intricate structure–property relationship elucidated with precision using high-resolution transmission electron microscopy.

**Supplementary Information:**

The online version contains supplementary material available at 10.1007/s40820-023-01290-4.

## Graphical abstract

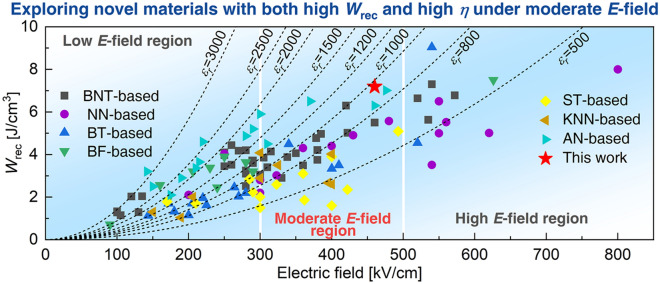

## Introduction

In contrast with supercapacitors, lithium–ion batteries, and fuel cells, dielectric ceramic capacitors have emerged as a focal point in pulse electrical devices owing to their remarkable power density, rapid charge–discharge response, and broad operating temperature range [1–3]. However, their current bottleneck resides in a relatively lower energy-storage (ES) density, impeding widespread integration into pulse electrical devices [4–6]. Consequently, there is a pressing need to engineer dielectric materials with heightened energy-storage performance (ESP). The assessment of a dielectric material's ESP typically involves parameters such as *W* (total ES density), *W*_rec_ (recoverable ES density), *W*_loss_ (dissipated ES density), and *η* (ES efficiency). These parameters are determined through the measurement of polarization versus electric field (*P*–*E*) curves and are calculated using the following equations [7]:

1 $$W = \mathop \int \limits_{0}^{{P_{\max } }} E\,{\text{d}}P$$

2 $$W_{{{\text{rec}}}} = \mathop \int \limits_{{P_{r} }}^{{P_{{{\text{max}}}} }} E\,{\text{d}}P$$

3 $$\eta = \frac{{W_{{{\text{rec}}}} }}{W}100\% = \frac{{W_{{{\text{rec}}}} }}{{W_{{{\text{rec}}}} + W_{{{\text{loss}}}} }}100\%$$

Here *P*_max_, *P*_*r*_, and *E* denote the maximum polarization, remnant polarization, and applied electric field (*E*-field), respectively. According to Eqs. (1–3), it is suggested that a dielectric material with a large Δ*P* (= *P*_max_–*P*_*r*_), high breakdown strength (BDS), and low *P*–*E* curve hysteresis would achieve high *W*_rec_ and *η* [8]. Dielectric materials are categorized into four types based on the characteristics of their *P*–*E* curves: linear dielectrics (LDs), normal ferroelectrics (FEs), antiferroelectrics (AFEs), and relaxor ferroelectrics (RFEs) [9]. LDs exhibit a linear relationship between *E*-field and induced polarization [10]. Despite this, their low *P*_max_ results in poor ESP. While FEs can achieve high *P*_max_, the elevated *P*_*r*_ and substantial *P*–*E* hysteresis lead to poor *P*_*r*_ and *η*. Lead-containing AFEs, although possessing higher *P*_*r*_, undergo an *E*-field-driven AFE-FE phase transition during electrical loading, resulting in lower *η* and a strain mutation at the critical *E*-field [11]. Consequently, RFEs outperform the other three types of dielectrics in terms of integrated ESP [12, 13].

**Fig. 1:**
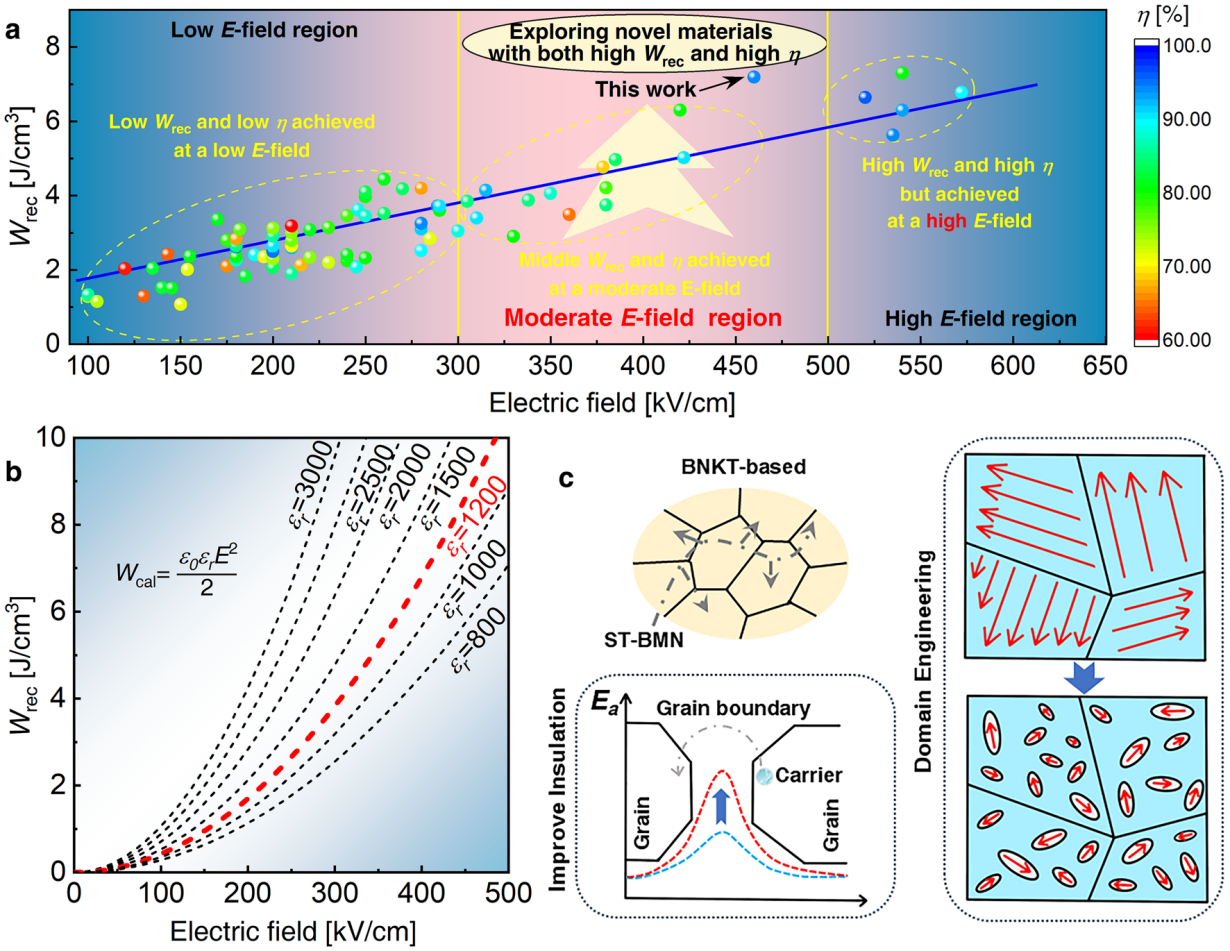
**a** ES density and characteristics under varying *E*-fields. **b** The theoretical correlation between *W*_rec_ and *E*-field. **c** Enhanced insulation performance and domain structure of BNKT through ST-BMN doping

The ESP of (Bi_0.5_Na_0.5_)TiO_3_ (BNT)-based bulk RFE ceramics has been extensively investigated in recent years [5, 6]. Figure 1a presents the *W*_rec_ of BNT-based ceramics plotted against the applied *E*-field, and Table S1 (refer to Supporting Information) summarizes the composition, maximum applied *E*-field (*E*_max_), *W*_rec_, and *η* for compared BNT-based bulk ceramics. In Fig. 1a, it is evident that *W*_rec_ generally increases with the applied *E*-field. Consequently, reducing permittivity (*ε*_*r*_) and enhancing BDS emerge as effective strategies for enhancing the ESP of BNT-based ceramics. While efforts to reduce permittivity and improve BDS are relatively comparable, two critical challenges have been overlooked for an extended period. (1) Although increasing BDS enhances material ES capacity, working with strong *E*-fields poses challenges for miniaturization and integration. Operation at high fields (> 500 kV cm^−1^) not only requires connecting the transformer system to the dielectric ES ceramic but also compromises the overall insulation performance of the system [14, 15]. This increases the system’s cost while reducing its safety. Finding ceramic capacitors with high *W*_rec_ and *η* under low *E*-fields (300 kV cm^−1^) can also be challenging. Therefore, it is imperative to explore innovative dielectric ceramics with high *W*_rec_ and *η* under moderate *E*-fields, as depicted in Fig. 1a. (2) Many studies have opted to delay saturation polarization to reduce *ε*_*r*_ and improve BDS [16, 17]. However, arbitrarily reducing *ε*_*r*_ can be detrimental. According to Eq. (4):

4 $$P = \varepsilon_{0} \varepsilon_{r} E$$

where *ε*_0_ is the vacuum permittivity. Extremely low *ε*_*r*_ diminishes macroscopic polarization, negatively impacting *W*_rec_ according to Eq. (2). Conversely, extremely high *ε*_*r*_ can lead to rapid polarization saturation and dielectric breakdown at low *E*-fields, reducing *η*. Therefore, understanding how to scientifically tailor *ε*_*r*_ is crucial. In Fig. 1b, we employed Eq. (5) to calculate the ESP of LD materials:

5 $$W_{{{\text{cal}}}} = \frac{{\varepsilon_{0} \varepsilon_{r} E^{2} }}{2}$$

where *W*_cal_ is the theoretical calculated value of *W*_rec_. As per Eq. (5), ES density is positively correlated with permittivity. However, RFEs are not linear dielectrics and exhibit substantial hysteresis, resulting in their *W*_rec_ typically being lower than *W*_cal_. BDS, on the other hand, is associated with permittivity, with higher permittivity making breakdown easier under low *E*-fields, leading to lower ESP. To achieve ultrahigh ESP under a moderate *E*-field of 500 kV cm^−1^, permittivity should be adjusted to around 1200, as shown in Fig. 1b.

Prior investigations suggest that lead-free bulk ES ceramics, comprising BaTiO_3_ (BT)-based [18, 19], (K_0.5_Na_0.5_)NbO_3_ (KNN)-based [20, 21], and BNT-based materials, exhibit high *P*_max_ values [5, 22–24]. Particularly within BNT-based materials, *P*_max_ can surpass 40 μC cm^−2^ [18, 25, 26]. However, the strong hybridization between the 6*s* orbital of Bi^3+^ ion and the 2*p* orbital of O^2–^ ion not only yields high *P*_max_ but also high *P*_*r*_ [27]. In our previous study, we introduced SrTiO_3_ (ST) into BNT-based ceramics, where Sr^2+^ entered the A-site, inducing A-site disorder [28]. Although this reduces *P*_max_, it significantly decreases *ε*_*r*_. In this investigation, we counterbalance the negative impact on polarization by incorporating Bi(Mg_2/3_Nb_1/3_)O_3_ (BMN). On the one hand, this is conducive to enhancing *P*_max_ by introducing Nb^5+^ ions at the B-site and increasing the content of Bi^3+^ ions at the A-site. Calculations indicate that the activation energy (*E*_*a*_) of grain boundaries rises with the increase in ST-BMN content. On the other hand, elevated *E*_*a*_ improves the material’s insulating capabilities, as depicted in Fig. 1c. Additionally, the introduction of different ions into the A-site and B-site effectively disrupts long-range order through local *E*-field fluctuations [29, 30]. Based on the findings of T. Karthik et al. and Jing et al., it can be shown that (Bi_0.5_Na_0.4_K_0.1_)TiO_3_ (referred to as BNKT) is situated in close proximity to the morphotropic phase boundary (MPB) [31, 32]. This particular positioning results in the manifestation of lower coercive field (*E*_*c*_) and bigger polarization when compared to BNT. BNKT ceramics located near the MPB can transform macroscopic ferroelectric domains into polar nanoregions (PNRs), fostering increased *η*, and enhancing the materials’ ESP. Consequently, under a moderate *E*-field, the (1–*x*)BNKT−*x*(2/3ST-1/3BMN) ceramics are anticipated to demonstrate superior ESP. Following the outlined strategy in Fig. 1, ST-BMN has been employed to modulate the domain, heighten the *E*_*a*_ of grain boundaries, and adjust the *ε*_*r*_ to approximately 1200. This approach aims to produce ceramic capacitors showcasing exceptional ESP within the range of moderate *E*-fields (300–500 kV cm^−1^). Ultimately, a breakthrough in the performance of BNT-based materials has been achieved, attaining a *W*_rec_ of 7.19 J cm^−3^ and an ultrahigh *η* of 93.9% under a moderate *E*-field of 460 kV cm^−1^, along with outstanding thermal stability spanning a temperature range of 30–140 °C.

## Experimental Section

### Sample Preparation

The conventional solid-state reaction technique, combined with the viscous polymer process, was employed for the fabrication of (1–*x*)BNKT−*x*(2/3ST-1/3BMN) ceramics, abbreviated as B-*x*SB, with *x* values of 0.35, 0.40, 0.45, and 0.50. The reactions and sintering were carried out at 880 and 1150 °C, respectively, for a duration of 2 h. Further details on the experimental procedures, encompassing sample preparation, structural characterization, and assessments of dielectric, ferroelectric, and ES properties, are provided in the Supplementary Information.

### Structural Characterization

The phase structure was thoroughly examined using X-ray diffraction (XRD, Panalytical, Cambridge, UK) in the range of 10°–120°, with a sweeping rate of 2° min^−1^, a working voltage of 45 kV, and a working current of 40 mA (operating conditions: Cu-K*α*, *λ* = 1.5418 Å). Prior to testing, the sintered ceramic samples were ground into powders and annealed at 500 °C for 4 h to alleviate internal stress. The refinement of the phase structure was conducted using the FULLPROF software package (version 2000). The microstructure of the sintered samples was visualized using a scanning electron microscope (SEM, Quanta, FEG 250, FEI, Hillsboro, USA). Dark-field images, selected area electron diffraction (SAED) patterns, and nanoscale high-resolution images were acquired using a specialized aberration-corrected transmission electron microscope (AC-TEM, Talos F200X, FEI, USA).

### Dielectric, Ferroelectric, and ES Properties Measurement

The temperature-dependent dielectric properties, encompassing *ε*_*r*_ and dielectric loss tangent (tan*δ*) of the investigated samples, were assessed using a multi-frequency LCR meter (E4980A, Agilent, Palo Alto, USA). The test parameters included a temperature range of 30–400 °C, a heating rate of 2 °C min^−1^, and test frequencies spanning 0.3–1000 kHz, respectively. *P*–*E* hysteresis loops and *J*–*E* curves for all samples were acquired using the Sawyer–Tower circuit (TF analyzer 2000, Aachen, Germany). Prior to the first-order reversal curve (FORC) and charge–discharge testing, a 2-mm diameter electrode was affixed to the surface of the samples, each with a thickness of approximately 100 μm. The direct ESP of the ceramic samples was evaluated using a charge–discharge testing system (CFD-003, TG Technology, Shanghai, China). *W*_dis_ and power density (*P*_*D*_) were computed employing the formulas ${W}_{{\text{dis}}}=R\int {i}^{2}\left(t\right){\text{d}}t/V$ and ${P}_{D}=E{I}_{{\text{max}}}/2S$, where *i*(*t*), *V*, *I*_max_, *R*, and *S* represent the time-dependent discharge current, effective volume, maximum discharge current, load resistance, and electrode area, respectively. FORC measurements were conducted using modulated triangle waveforms with a standard ferroelectric testing device (TF Analyzer 2000, aixACCT, Aachen, Germany). The Preisach density representing the distribution of FORC data was calculated from the descending segment of the primary hysteresis loop using a differential approach, as described by the following equation [33]:

5 $$\rho_{{{\text{FORC}}}}^{ - } \left( {E,E_{r} } \right) = \frac{{\partial^{2} P_{{{\text{FORC}}}}^{ - } \left( {E,E_{r} } \right)}}{{\partial E_{r} \partial E}}$$

Here ${P}_{{\text{FORC}}}^{-}$ denotes the polarization in the FORC analysis, *E*_*r*_ represents the reversal electric field, *E* is the actual applied electric field, and ${\rho }_{{\text{FORC}}}^{-}$ signifies the Preisach density of the FORC distribution. The presence of the minus sign indicates that the FORC analysis commences from the descending branch of the primary hysteresis loop.

## Results and Discussion

### XRD Structural Characterization

**Fig. 2:**
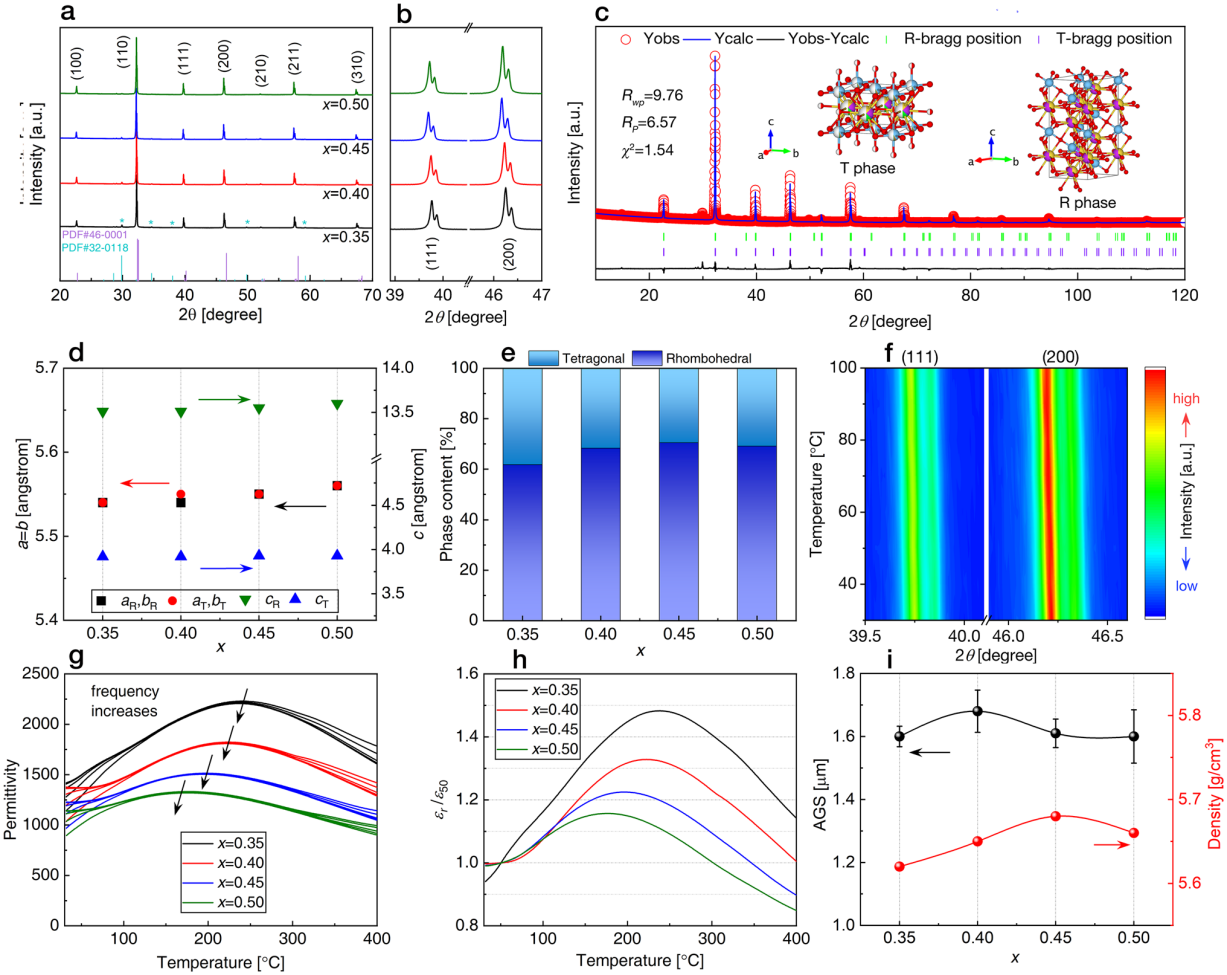
**a** XRD pattern of ceramics with varying *x* (0.35, 0.40, 0.45, and 0.50) in the 20°–70° range. **b** Enlarged views of the diffraction peaks at 39°–40.5° and 45.5°–47°. **c** Fitted XRD pattern for *x* = 0.50 ceramic powders. **d** Variation of lattice parameters with *x* for B-*x*SB ceramics. **e** Proportion variation of the R and T phases for different compositions. **f** Temperature evolution of XRD patterns for *x* = 0.50 ceramic from 30 to 100 °C at selected angles of 39.5°–40° and 46°–46.5°. **g** Temperature-dependent *ε*_*r*_ and tan*δ* for *x* = 0.35, 0.40, 0.45, and 0.50 at measurement frequencies of 0.3, 1, 10, 100, and 1000 kHz, ranging from 30 to 400 °C. The long arrow indicates the direction of increasing measurement frequency. **h** *ε*_*r*_/*ε*_50_ as a function of temperature measured at 1 kHz, where *ε*_50_ was measured at 50 °C, and *ε*_*r*_ was measured from 30 to 400 °C. **i** AGS and density variations with *x* for B-*x*SB ceramics

The XRD patterns of B-*x*SB ceramic powders, with scanning angles 2*θ* ranging from 20° to 70°, are presented in Fig. 2a. All component diffraction peaks align with the BNT PDF #46-0001. Additionally, traces of a second phase were detected, with diffraction peaks potentially coinciding with the PDF #32-0118 of Bi_2_Ti_2_O_7_, consistent with the previous findings [34, 35]. In Fig. 2b, the diffraction peaks at 39°–40.5° and 45.5°–47° are magnified. For *x* > 0.45, the (111) and (200) peaks shift toward lower angles, while others remain unchanged. To gain a deeper understanding, Rietveld refinement was employed to identify crystal symmetry and lattice characteristics. Table S2 and Fig. S1 summarize the fitting parameters. All B-*x*SB ceramic samples exhibit a coexistence of tetragonal (*T*) phase with *P*4*bm* space group and rhombohedral (*R*) phase with *R*3*c* space group, as demonstrated by the Rietveld refinement of B-0.5SB in Fig. 2c. Figure 2d shows that when *x* ≥ 0.45, lattice parameters increase slightly, corresponding to the shift of (111) and (200) peaks.

ST and BMN form a solid solution with BNKT, allowing Sr^2+^ and Bi^5+^ to enter the A-site, according to our composition design. At a coordination number (CN) of 12, the ion radii of Sr^2+^ and Bi^5+^ are 144 and 130 pm, respectively [36]. Their radii in BNKT are between those of Bi^5+^ (130 pm), Na^+^ (139 pm), and K^+^ (164 pm). Therefore, A-site substitution does not result in an apparent lattice expansion. Both Mg^2+^ and Nb^5+^ ions enter the B-site simultaneously. With CN = 6, the radius of Ti^4+^ is 60.5 pm, while Nb^5+^ and Mg^2+^ are 64 and 72 pm, respectively. The radii of Nb^5+^ and Mg^2+^ ions are slightly larger than Ti^4+^. When the doping level approaches 45% and 50%, Mg^2+^ replaces less than 10% of Ti^4+^ [36]. Therefore, when the doping amount increases to 45%, the crystal lattice slightly expands. However, when the doping quantity is too low, it does not generate an apparent lattice expansion, consistent with the previous results by Wang et al. [37] and Sun et al. [38]. As *x* increases from 0.35 to 0.50, the content of *R* phase increases somewhat and then declines, as shown in Fig. 2e. The *T* phase to *R* phase ratio remains nearly constant. The preservation of a strong polar phase is beneficial for maintaining macroscopic polarization. Figure 2f depicts the evolution of (111) and (200) diffraction peaks as the temperature rises from 30 to 100 °C. Due to the thermal expansion of the crystal lattice, these two peaks shift only toward lower angles with increasing temperature. The shape of these two peaks remains unchanged across the temperature range, indicating the coexistence of *R* and *T* phases from room temperature (RT) to 100 °C.

### Dielectric Properties

The phase evolutions of the B-*x*SB ceramics were further assessed through their dielectric properties. The temperature-dependent *ε*_*r*_ of B-*x*SB ceramic samples, measured at various frequencies, is illustrated in Fig. 2g. In this temperature range, each composition exhibits two dielectric anomalies, a common occurrence in BNT-based systems [12, 28, 32, 39]. The dielectric anomaly with high dielectric dispersion on the left is attributed to a normal dielectric relaxation, while the broad dielectric peak with low dielectric dispersion on the right is associated with a diffuse phase transition caused by the coexistence of *R* and *T* phases [40, 41] or a transformation from low-temperature PNRs to high-temperature PNRs [28, 42–44]. The dielectric anomaly peak on the left shifts toward higher temperatures with increasing frequency, indicative of a typical RFE characteristic [45, 46]. The temperature (*T*_m_) at which the maximum permittivity (*ε*_m_) occurs demonstrates a clear shift toward lower values as x increases from 0.35 to 0.5. This behavior can be attributed to the disruption of the long-range ferroelectric order and the simultaneous increase in PNRs content. Notably, *ε*_*r*_ decreases with increasing BMN concentration, reaching the lowest value for the *x* = 0.50 composition. The curves of *ε*_*r*_/*ε*_50_ (where *ε*_50_ is the *ε*_*r*_ measured at 1 kHz and 50 °C) with increasing temperature are depicted in Fig. 2h. The *x* = 0.50 composition exhibits the lowest and most stable *ε*_*r*_/*ε*_50_, signifying superior temperature stability. Due to its low *ε*_*r*_ and excellent temperature stability, this composition holds great appeal for dielectric capacitor applications.

### Surface Microstructures

**Fig. 3:**
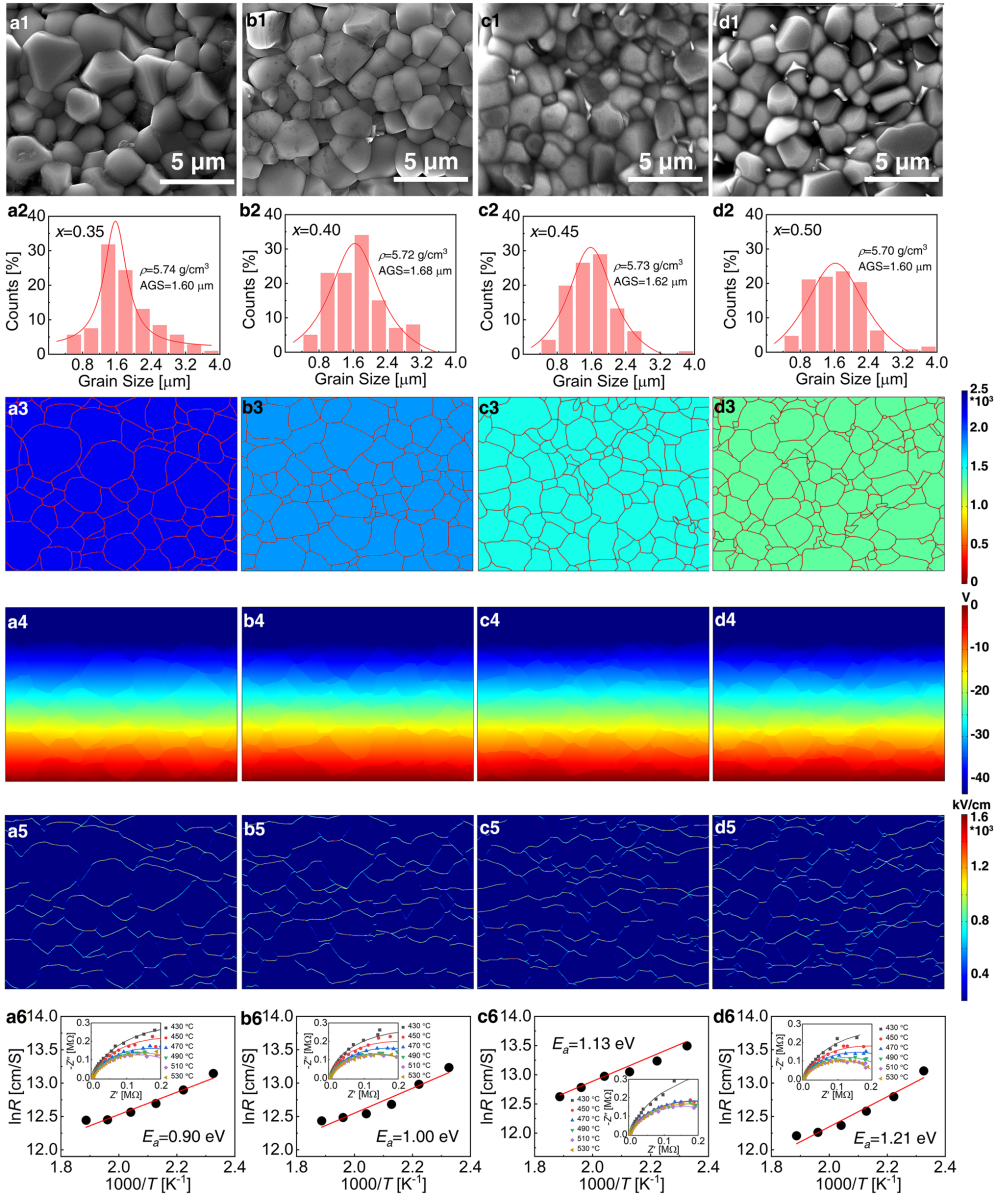
SEM images of surface morphologies for compositions **a1** *x* = 0.35, **a2** *x* = 0.40, **a3** *x* = 0.45, and **a4** *x* = 0.50 ceramics. **a2**–**d2** Grain size distribution. **a3**–**d3** Intensity mapping of *ε*_*r*_. **a4**–**d4** Distribution of electric potentials. **a5**–**d5** Local *E*-field distribution. **a6**–**d6** Relationship between temperature (T) and grain boundary resistance, with resistance spectra shown in the insets

SEM was employed to unveil the microstructures of B-*x*SB ceramics. In Fig. 2i, the average grain size (AGS) and density of B-*x*SB ceramics are depicted. The AGS and density exhibit slight variations with increasing BMN concentration, measuring between 1.60 and 1.68 μm and between 5.62 and 5.68 g cm^−3^, respectively. The surface morphologies of B-*x*SB ceramic samples, shown in Fig. 3a1–d1, reveal dense structures with minimal porosity. While no obvious second phase is observed in Fig. 3a1–b1, a small amount of second phase is apparent in Fig. 3c1–d1. As suggested by the XRD data, these second-phase materials are highly correlated to an oxide formed by the precipitation of Bi and Ti. This phenomenon of Bi precipitation is a recurring observation in BNT-based materials. Despite our concerted efforts to mitigate Bi precipitation during the sintering process through various methods, a minor amount of precipitation remains. It is important to note that similar observations of Bi precipitation have been documented in prior studies focusing on BNT-based materials [34, 35]. This phenomenon seems to be a common characteristic within this class of materials. The statistical diagrams of grain size distribution, presented in Fig. 3a2–d2, indicate that all grain size distributions follow a normal distribution.

Combining surface morphology and permittivity, we simulated the applied *E*-field to investigate potential distribution and local *E*-field distribution. SEM was utilized to individually portray grains and grain boundaries, and the *ε*_*r*_ intensity map is provided in Fig. 3a3–d3. Figure 3a4–d4 illustrates the potential distribution after simulating an external *E*-field of 150 kV cm^−1^ downward. It is evident that as *x* increases, the potential difference between grains decreases significantly. As shown in Fig. 3a5–d5 (local *E*-field distribution), the regions represented by white circles exhibit the greatest potential difference and are more likely to experience breakdown, particularly at grain boundaries, elucidating the ceramics' susceptibility to grain boundary breakdown. When *x* = 0.50, the breakdown resistance performance improves. Moreover, the impedance of B-*x*SB ceramics was measured between 430 and 530 °C, as depicted in Fig. 3a6–d6. The grain boundary resistance was calculated using the (CR)(CR) model fitting, and *E*_*a*_ was determined using the Arrhenius formula [47]:

6 $$\sigma = A \cdot e^{{\frac{{ - E_{a} }}{{k_{B} \cdot T}}}}$$

where *σ* is conductivity, *A* is a constant, *k*_B_ is the Boltzmann constant, and *T* is temperature. Taking the derivative on both sides of the formula yields *E*_a_. The results indicate that as ST-BMN concentrations increase, *E*_*a*_ rises from 0.90 eV (*x* = 0.35) to 1.21 eV (*x* = 0.50). The larger *E*_*a*_ inhibits carriers from crossing grain boundaries, effectively enhancing material insulation. These findings lay the groundwork for improving BDS.

### Nanoscale Microstructures

**Fig. 4:**
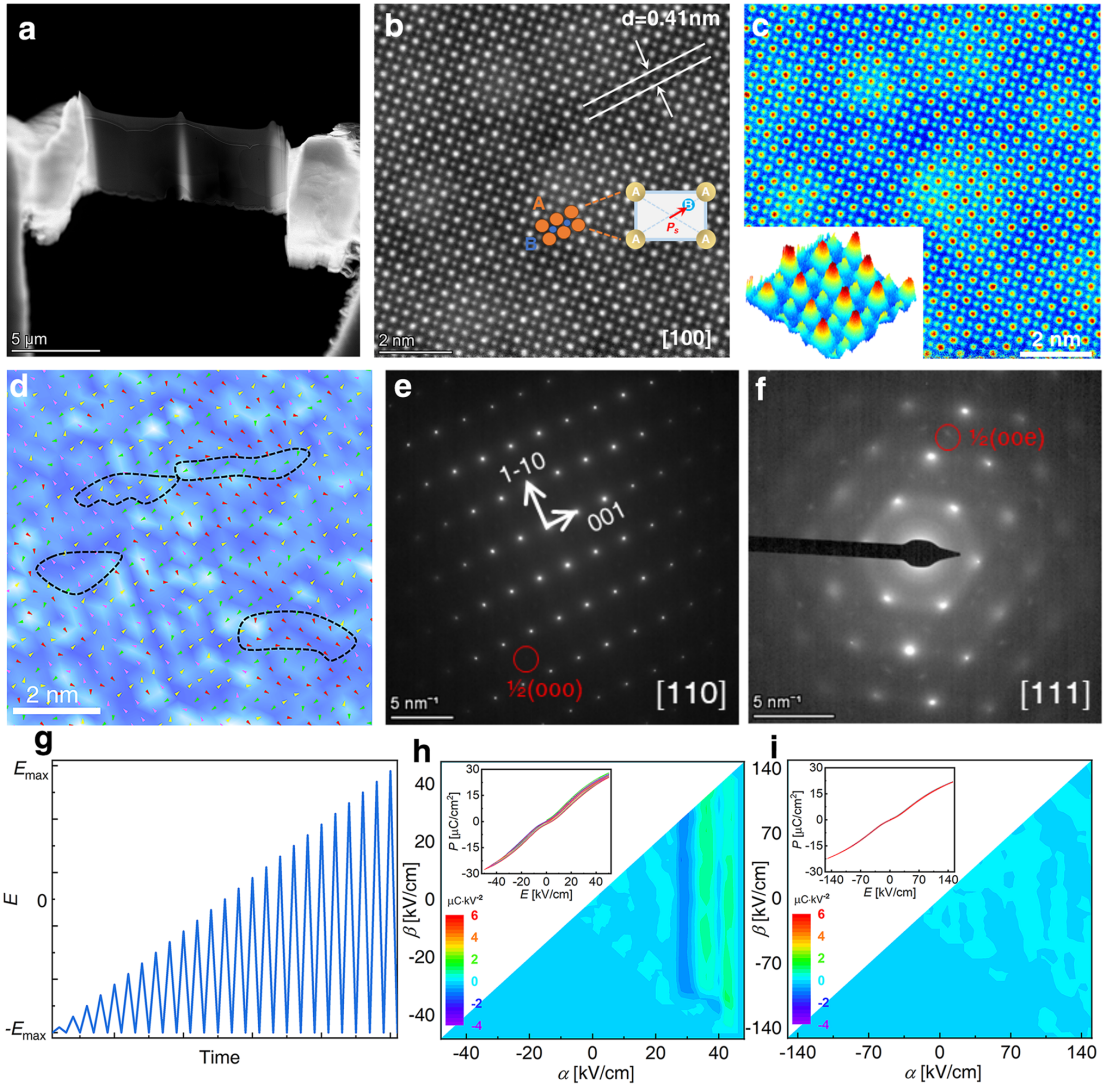
**a** Bright-field and **b** high-resolution TEM image of BSB-0.5 ceramic. **c** Redistribution of brightness based on the RGB values of **b**. **d** The polarization vector, calculated from cation displacement on the B-site, superimposed on the polarization intensity distribution, with relative polarization intensity expressed through the brightness and saturation of the background color. SAED patterns along **e** [110]_pc_ and **f** [111]_pc_. **g** FORC test method. Evolution of FORC distributions for **h** *x* = 0.35 and **i** *x* = 0.50

The nanoscale microstructures of the *x* = 0.50 composition were meticulously examined using an AC-TEM. The bright-field image of a sample prepared through focused ion beam (FIB) is presented in Fig. 4a. High-resolution (HR)-TEM images of the *x* = 0.50 composition are detailed in Fig. 4b, where the distribution of cations is reflected by the imaging brightness. At the A-site, the brightness of Na^+^, Bi^3+^, K^+^, and Sr^2+^ cations is comparatively high, while at the B-site, the brightness of Nb^5+^, Mg^2+^, and Ti^4+^ cations is low. The brightness distribution is further illustrated in Fig. 4c insert, with high brightness in red and low brightness in blue. Figure 4c distinctly reveals the distribution of cations on the B-site (in yellow and green). The coordinates of the A-site atoms are extracted using the red region’s coordinates, and the theoretical coordinates of B-site atoms are calculated. Subsequently, the actual coordinates of B-site atoms are extracted based on the coordinates of the yellow and green regions, facilitating the simulation of the spontaneous polarization vector in each cell, as depicted in Fig. 4d. The background in Fig. 4d is an intensity map of polarization, showcasing the random orientation of spontaneous polarization vectors in each cell. Different colors represent polarization vectors in different directions, a characteristic of RFE, forming the basis for unique properties and the chemical foundation for the existence of PNRs. The strong polar and weak polar regions, depicted in dark blue and light blue backgrounds, respectively, are randomly distributed, and the dipoles within the white circle exhibit the same orientation, indicating PNRs. All PNRs have a diameter of less than 5 nm, indicating effective refinement by local *E*-field fluctuations, which enhances the ESP of the material [48–50].

Figure 4e and f shows selected area electron diffraction (SAED) patterns along [110]_pc_ and [111]_pc_, where pc denotes pseudo-cubic. The presence of 1/2(ooo) superlattice points in [110] indicates the R phase, while the presence of 1/2(ooe) superlattice points in [111] indicates the T phase [51]. These patterns confirm that the composition with *x* = 0.50 possesses a phase structure consisting of coexisting R and T phases, consistent with the Rietveld fitting result of XRD. While TEM can reveal domain morphologies at the nanometer scale, it typically provides static results. In contrast, piezoresponse force microscopy (PFM) elucidates polarization switching behavior at the micrometer scale but faces challenges in capturing dynamic details, especially in relaxor ferroelectrics [52]. In these materials, PNRs rather than macroscopic domains respond to external driving fields. The switching behavior of individual PNR may vary due to local electric field variations, and historical responses can influence polarization switching behavior. FORC analysis is a valuable technique that can detect a range of residual states dependent on field history. It effectively represents the reaction of local structures in the presence of a uniform electric field and provides a statistical outcome of polarization switches in *P–E* loops. This approach incorporates spatial and historical polarization dynamics, offering direct insights into microstructures [53, 54]. FORCs of *x* = 0.35 and 0.50 ceramics were examined to illustrate macroscopically that in a sample with *x* = 0.50, all domains are converted into microscopic PNRs. Figure 4g and h–i depicts the principle and FORCs of ceramics with *x* = 0.35 and 0.50, respectively. In simpler terms, FORC is employed to quantify the number of polarization reversals: A higher value indicates more reversals, while a lower value signifies fewer reversals. The motion of domain barriers is recorded by tracking changes in domains through polarization reversals. In low *E*-fields, the low number of ferroelectric phases and the strong nonlinear polarization behavior of *x* = 0.35 ceramics at 30 kV cm^−1^ indicate the inversion of ferroelectric domains and the movement of domain walls. This significant hysteresis is a consequence of weak ESP. Even when the sample with *x* = 0.50 is loaded to 150 kV cm^−1^, there is no high-intensity distribution, indicating that ferroelectricity is diluted, RFE features are enhanced, and hysteresis is diminished. This is consistent with the exclusive discovery of tiny PNRs by TEM.

### Ferroelectric and ES Performance

**Fig. 5:**
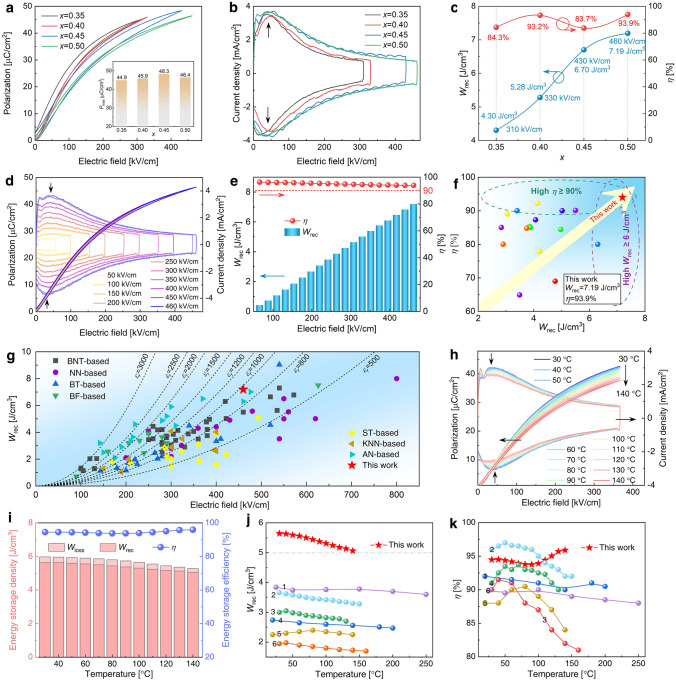
**a** *P*–*E* loops and **b** the corresponding current density–electric field (*J*–*E*) curves for *x* = 0.35, 0.40, 0.45, and 0.50, measured at RT, 10 Hz, and various *E*-fields. **c** *W*_rec_ and *η* of *x* = 0.35, 0.40, 0.45, and 0.50 ceramics. BDS is indicated next to the data. **d** *P*–*E* loops and corresponding *J*–*E* curves for *x* = 0.50 composition measured at RT and 10 Hz. **e** *W*_rec_ and *η* of *x* = 0.50 composition. **f** Comparison of *W*_rec_ and *η* for BSB-0.50 and other BNT-based bulk ceramics. **g** Comparison of *W*_rec_ for B-0.5SB ceramic with other bulk ceramics. **h** *P*–*E* loops and *J*–*E* curves of B-0.5SB ceramic measured at 10 Hz and 366 kV cm^−1^. Temperature increases from 30 to 140 °C in steps of 10 °C. **i** *W*_loss_, *W*_rec_, and *η* calculated from the corresponding *P*–*E* loops of B-0.5SB. Temperature-dependent *W*_rec_ **j** and *η* (k) of B-0.5SB compared with other BNT-based bulk ceramics

Subsequently, the ferroelectric and ES properties of B-*x*SB ceramic samples were thoroughly assessed. Figure 5a illustrates the unipolar *P*–*E* hysteresis loops of B-*x*SB samples with *x* = 0.35, 0.40, 0.45, and 0.50. Owing to the decrease in *ε*_*r*_, the *E*_max_ that can be applied to the sample before electrical breakdown increases with the rising values of *x*. For B-*x*SB samples with *x* ranging from 0.35 to 0.50, the respective *E*_max_ values are 310, 330, 430, and 460 kV cm^−1^ at a test frequency of 10 Hz. All B-*x*SB samples exhibited slim hysteresis loops, characteristic of RFE ceramics [9]. As *x* increases, the *P*–*E* loops become slimmer. Corresponding *J*–*E* curves are presented in Fig. 5b, revealing two distinct current peaks at 50 kV cm^−1^. The RFE nature of B-*x*SB samples disrupts long-range ferroelectric order, resulting in the appearance of PNRs. When an applied *E*-field is present, PNRs become oriented but are unable to grow into macrodomains and return to random orientation when the *E*-field drops from *E*_max_ to zero. Consequently, the *J*–*E* curves exhibit twin peaks at low *E*-fields. Given the ruled-out AFE nature of BNT-based solid solutions [55, 56], the very slim *P*–*E* loops combined with double current peaks in *J*–*E* curves should be described as an AFE-like RFE (AL-RFE) characteristic [8, 57], rather than a relaxor AFE characteristic [58–60]. Furthermore, Fig. 5b indicates that these two current maxima are roughly symmetric and remain fairly constant as *x* increases. It is noteworthy that the *P*_max_ determined at the same *E*_max_ gradually decreases as *x* increases from 0.35 to 0.50, owing to the decoupling of A-O and B–O bonds [61]. However, the Pmax of B-*x*SB ceramic samples evaluated at their respective *E*_max_ exhibits negligible variation versus *x*. This suggests that the loss of *P*_max_ due to the decoupling effect can be compensated for by increasing *E*_max_. Figure 5a also reveals that the *P*_*r*_ has decreased from 2.64 (*x* = 0.35) to 0.015 (*x* = 0.50) μC cm^−2^, allowing the Δ*P* and *W*_rec_ to grow. *W*_rec_ and *η* are determined using the unipolar *P*–*E* loops shown in Fig. 5a. When the *E*_max_ increases by 48% from 310 kV cm^−1^ (*x* = 0.35) to 460 kV cm^−1^ (*x* = 0.50), the *W*_rec_ increases by 67% from 4.30 J cm^−3^ (*x* = 0.35) to 7.19 J cm^−3^ (*x* = 0.50), while the *η* of B-*x*SB ceramic samples remains around 90%. This result indicates that the *x* = 0.50 composition has superior ESP than the other three compositions. Consequently, Fig. 5d depicts the comprehensive* P*–*E* loops and corresponding *J*–*E* curves of the *x* = 0.50 composition measured from 50 to 460 kV cm^−1^. Under a small *E*-field, the *J*–*E* curves exhibit a rectangular form. Due to the *E*-field-induced RFE-to-polar phase transition [27, 56], double current maxima are observed in the *J*–*E* curves when the *E*-field exceeds 150 kV cm^−1^. The field corresponding to these two maximum values of current scarcely fluctuates, demonstrating that even if the *E*-field is increased to *E*_max_, the critical *E*-field for RFE-to-polar phase transition will remain unchanged. More crucially, as the *E*-field grows, the *P*–*E* loops remain slim and do not broaden, indicating that no conductive process has been activated, and a high *η* can be achieved. Figure 5e depicts the *W*_rec_ and *η* of the *x* = 0.50 composition under various *E*-fields. The *W*_rec_ grows monotonously and reaches an impressive value of 7.19 J cm^−3^ at 460 kV cm^−1^, whereas the *η* shows very little reduction and still maintains a very high value of 94% at the same *E*-field.

Figure 5f compares the ESP of the *x* = 0.50 ceramic with that of other BNT-based bulk ceramics [25, 62–78]. Details regarding the composition, *E*_max_, *W*_rec_, and *η* of the compared BNT-based bulk ceramics are summarized in Table S3. Clearly, the ESP of the *x* = 0.50 composition surpasses that of existing BNT-based ceramics. Generally, increasing the *E*-field improves *W*_rec_. However, in the high *E*-field region, it appears that augmenting the electric field does not directly enhance *W*_rec_. Although the *E*_max_ values of 0.8BNT-0.2Sr(Nb_0.5_Al_0.5_)O_3_ [74] and 0.85[0.7BNT–0.3(Bi_0.1_Sr_0.85_)TiO_3_]–0.15KNbO_3_ [72] ceramics are 520 and 569 kV cm^−1^, respectively, their *W*_rec_ values are 6.64 and 5.63 J cm^−3^, which are inferior to the *W*_rec_ of our ceramic. Conversely, even with high *W*_rec_ values of 7.02 and 6.3 J cm^−3^ at 390 and 420 kV cm^−1^, the *η* values of 0.78BNT-0.22NaNbO_3_ [65] and 0.85(0.94BNT-0.06BT)-0.15Bi(Mg_2/3_Nb_1/3_)O_3_ [25] ceramics, at 85% and 80% respectively, are significantly lower than our ceramic (94%). Consequently, we achieve a comprehensive improvement in ESP with both high *W*_rec_ and high *η* in the *x* = 0.50 ceramic sample. In Fig. 5g, the ESP of the *x* = 0.50 ceramic is compared to that of various representative dielectric ceramic materials, including linear ST-based systems, AFE AgNbO_3_ (AN)-based and NaNbO_3_ (NN)-based systems, and RFE BT-based, KNN-based, BF-based, and BNT-based systems. Details of the composition, *E*_max_, *W*_rec_, and *η* of the compared systems, are summarized in Table S4. It is evident that linear ST-based systems often have low *W*_rec_ (< 4 J cm^−3^), but AFE AN-based and NN-based systems exhibit higher *W*_rec_ (4–7 J cm^−3^) and low *η* (50–90%). In contrast, RFE BT-based and KNN-based systems show medium *W*_rec_ (2–4 J cm^−3^) and fluctuating *η* (50–95%) under a moderate *E*-field. Although some BNT-based systems achieve higher *W*_rec_ and very high η (> 90%), the *E*_max_ has approached 520–600 kV cm^−1^. In contrast, our *x* = 0.50 composition exhibits high *W*_rec_ (> 7 J cm^−3^) and high *η* (94%) simultaneously at a moderate *E*_max_ (460 kV cm^−1^), indicating a comprehensive improvement in ESP. These findings demonstrate that by manipulating *ε*_*r*_, we were able to create B-*x*SB ceramics with exceptional ESP in a moderate *E*-field. As a result, it has emerged as one of the most promising candidate materials for pulse ES ceramic devices.

Temperature stability is a critical criterion for pulse ES ceramic devices [79, 80]. The temperature stability of the ESP for the *x* = 0.50 composition was evaluated using temperature-dependent *P*–*E* and *J*–*E* curves, as illustrated in Fig. 5h. These curves were measured at 365 kV cm^−1^ and 10 Hz, with temperatures ranging from 30 to 140 °C. Typically, in RFEs, *P*_*r*_ and *E*_*c*_ increase with rising temperature due to thermal activation of defects, which is detrimental to the stability of *W*_rec_ and *η* [7, 9, 81]. As the temperature increases, the *P*–*E* loops remain thin, and *P*_max_ decreases slightly from 42.7 to 37.0 μC cm^−2^. The two current peaks indicated by the black arrows show almost no movement with rising temperature. These findings suggest that the ESP in the *x* = 0.50 composition exhibits good thermal stability. Despite a slight reduction in temperature, the *W*_rec_ of the *x* = 0.50 composition decreases from 5.64 J cm^−3^ at 30 °C to 5.06 J cm^−3^ at 140 °C, a 10% reduction, as shown in Fig. 5i. Because the *P*_*r*_ and *E*_*c*_ of the *x* = 0.50 composition remained reasonably steady, its *η* is extraordinarily high, with minimal variations ranging from 93.9 to 95.9%. The temperature-dependent ESP of the *x* = 0.50 composition is compared to other previously reported BNT-based ceramics in Fig. 5j–k [62, 64, 65, 74, 82, 83]. The constituents of these BNT-based ceramics are summarized in Table S5 (see Supplementary Information). The *W*_rec_ in those BNT-based ceramics typically declines to some extent as temperature rises, with none exceeding 4 J cm^−3^. In contrast, the *W*_rec_ of our *x* = 0.50 composition is significantly higher than the values of various BNT-based ceramics in the 30–140 °C temperature range, surpassing 5 J cm^−3^ at this temperature range, as shown in Fig. 5j. Furthermore, upon comparing the *η*, it is evident that the *η* of the *x* = 0.50 composition remains around 95%. Even with rising temperature, the trend is upward, as illustrated in Fig. 5k. In conclusion, in addition to the ultrahigh ESP at RT, the ESP of the *x* = 0.50 composition demonstrates ultrahigh *W*_rec_ and exceptional *η* over a wide temperature range, outperforming other BNT-based ceramics.

### Charge–Discharge Performance

**Fig. 6:**
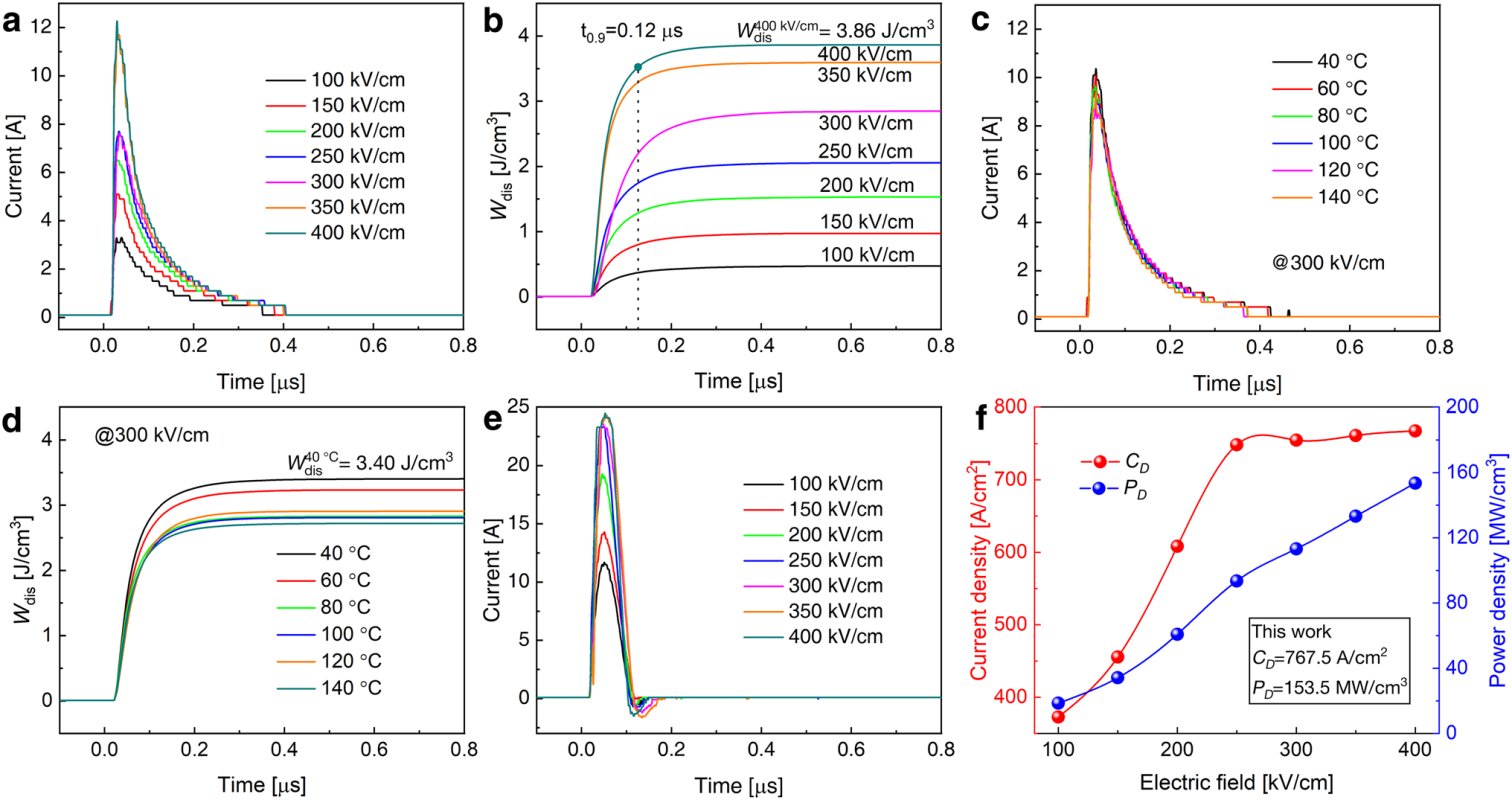
Pulsed overdamped discharging properties of B-0.5SB bulk ceramics, illustrating **a** current curves and **b** *W*_dis_ at RT and various electric fields. Temperature-dependent pulsed overdamped discharging properties of B-0.5SB bulk ceramics, depicting **c** current curves and **d** *W*_dis_ at 300 kV cm^−1^ as the temperature increases from 40 to 140 °C in intervals of 20 °C. Pulsed underdamped discharging properties of B-0.5SB bulk ceramics, exhibiting **e** current curves, and **f** *C*_*D*_ and *P*_*D*_ as functions of the electric field at RT

Finally, we evaluate the charge–discharge performance of the *x* = 0.50 composition, a pivotal criterion for pulse capacitor devices [84]. The sample thickness is 100 μm, and the electrode area is 3.14 mm^2^. Charge–discharge tests employ a 100-Ω resistor. A dielectric ceramic with rapid charge/discharge characteristics is well-suited for pulse power supply. Room temperature electric current versus time (*I*–*t*) curves measured in an overdamped discharge mode are presented in Fig. 6a. The *I*_max_ increases with the escalating electric field. *W*_dis_ attains its peak value of 3.89 J cm^−3^ under 400 kV cm^−1^, as depicted in Fig. 6b. The time at which *W*_dis_ releases 90% of its total energy, often denoted as *t*_0.9_, is crucial for calculating discharge speed. At 100 kV cm^−1^, the *t*_0.9_ for the *x* = 0.50 composition is approximately 0.21 μs, diminishing with increasing electric field. At 400 kV cm^−1^, *t*_0.9_ is less than 0.12 μs. Figure 6c illustrates the temperature-dependent *I*–*t* curves from 40 to 140 °C, determined at overdamped discharge mode under an *E*-field of 300 kV cm^−1^. The *t*_0.9_ remains below 0.13 μs at all measured temperatures, as demonstrated in Fig. 6d. The *I*–*t* curves of underdamped discharge at different *E*-fields are presented in Fig. 6e, and the calculated current density (*C*_*D*_) and power density (*P*_*D*_) are displayed in Fig. 6f. The discharge of the *x* = 0.50 composition is confirmed to be complete after only two oscillations. At 400 kV cm^−1^, *C*_*D*_ and *P*_*D*_ are exceedingly high at 767.5 A cm^−2^ and 153.5 MW cm^−3^, respectively. These features indicate that the *x* = 0.50 composition ceramics exhibit significant potential for use in pulse power devices [7, 82].

## Conclusions

In summary, we propose an approach to achieve optimal ESP under constrained *E*-field conditions by tailoring the *ε*_*r*_. Ultimately, the *x* = 0.50 composition demonstrates an extraordinary *η* of 93.8% and an impressive *W*_rec_ of 7.19 J cm^−3^ at a moderate *E*-field. The temperature-dependent ESP of the *x* = 0.50 composition is also notably robust. Across the temperature range of 30–140 °C, *W*_rec_ consistently exceeds 5 J cm^−3^ with high efficiency (above 95%). In contrast with other BNT-based bulk ceramic capacitors, the B-0.5SB ceramic, with its exceptional ESP, undoubtedly stands out as a promising candidate for future ES devices. Moreover, the B-0.5SB ceramic exhibits an ultrahigh current density and power density, simulating real-world application scenarios. Although this study employed linear dielectrics as a model and acknowledges some discrepancies between research results and theoretical conclusions, it addresses a crucial research gap concerning the attainment of high ESP in a restricted *E*-field. To conclude, this strategy, involving the modification of *ε*_*r*_, enhancement of insulating properties, and incorporation of domain engineering, proves effective. It is anticipated that these findings will contribute to and guide future research and development endeavors in the realm of ceramic capacitors.

## Supplementary Information

Below is the link to the electronic supplementary material.

Supplementary file1 (PDF 1034 kb)
