# Supplementary material for: Moderate Fields, Maximum Potential: Achieving High Records with Temperature-Stable Energy Storage in Lead-Free BNT-Based Ceramics
Source: Nanomicro Lett. 2024 Jan 18;16:91. doi: 10.1007/s40820-023-01290-4 (PMC10796886; doi:10.1007/s40820-023-01290-4)
Supplement: Supplementary file 1 — Supplementary file1 (PDF 1034 kb) [file 40820_2023_1290_MOESM1_ESM.pdf]

Supporting Information for

Moderate Fields, Maximum Potential: Achieving High Records with Temperature-Stable Energy Storage in Lead-Free BNT-Based Ceramics

Wenjing Shi[1], Leiyang Zhang[1], Ruiyi Jing[1], Yunyao Huang[1], Fukang Chen[2], Vladimir Shur[3], Xiaoyong Wei[1], Gang Liu[2,*], Hongliang Du[4,*], Li Jin[1,*]

[1] Electronic Materials Research Laboratory, Key Laboratory of the Ministry of Education, School of Electronic Science and Engineering, Xi'an Jiaotong University, Xi'an 710049, P. R. China

[2] School of Materials and Energy, Southwest University, Chongqing 400715, P. R. China

[3] School of Natural Sciences and Mathematics, Ural Federal University, Ekaterinburg, 620000, Russia

[4] Multifunctional Electronic Ceramics Laboratory, College of Engineering, Xi'an International University, Xi'an 710077, P. R. China

*Corresponding authors. E-mail: liugang13@swu.edu.cn (Gang Liu), duhongliang@126.com (Hongliang Du), ljin@mail.xjtu.edu.cn (Li Jin)

S1 Experimental Method

Sample Preparation: Convectional solid-state reaction method was used for the preparation of the (1–*x*)BNKT-*x*(2/3ST-1/3BMN) (BNKT-*x*SB) ceramics. High-purity row powder materials, which were manufactured by Sinopharm (Shanghai, China), including Na_2CO_3 (99.8%), K_2CO_3 (99%), Bi_2O_3 (99%), TiO_2 (98%), $SrCO_3$ (99%), MgO (98.5%), and Nb_2O_5 (99.5%) were weighed and mixed with ethanol, and then ball-milled for 24 h. The mixed slurry was dried at 120 °C and calcined at 880 °C for 2 h. After calcination, the pre-synthesized powders were ball-milled and dried again. Subsequently, 5 wt.% polyvinyl alcohol (PVA) solution and moderate deionized water were added to the synthesized powders to form a viscous gelatinous mixture, 5 wt% PVA to deionized water to powder mass ratio 1:4:5, and then repeated twin-rolled for 2 h for the ultra-thin green ceramic sheets around 110 μm. Finally, the sheets were punched into a lot of green sample discs with a diameter of 13 mm. The binder was burned out at 600 °C for 4 h and discs were sintered at 1150 °C for 2 h.

S2 Supplementary Figures and Tables

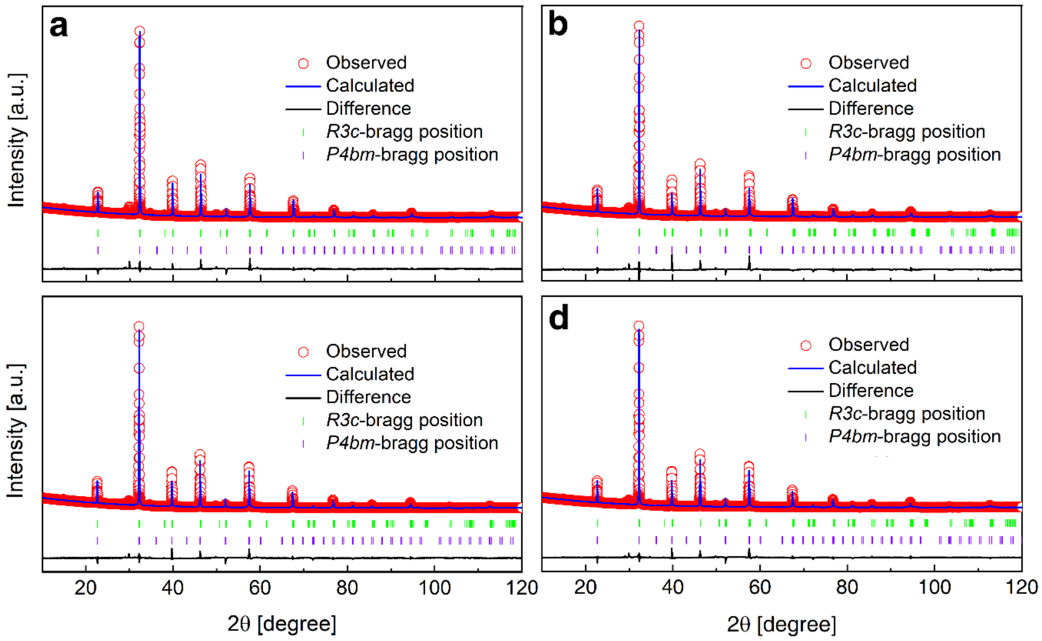

Fig. S1 Fitted X-ray powder diffraction pattern of B-*x*SB ceramics powders of (**a**) *x*=0.35, (**b**) *x* =0.40, (**c**) *x* =0.45 and (**d**) *x* =0.50 in range of 10-120°

Table S1 Energy storage properties of this work compared with other BNT-based bulk ceramics

Composition	E_{max} (kV/cm)	W_{rec} (J/cm^3)	η (%)	Refs.
[0.94($Bi_{0.5}Na_{0.5}$)TiO_3-0.06$BaTiO_3$]-0.02Ba($Sn_{0.70}Nb_{0.24}$)O_3	100	1.28	74	[S1]
0.7$Na_{0.5}Bi_{0.5}TiO_3$-0.3$Sr_{0.7}Bi_{0.2}TiO_3$-0.05Mn	100	1.33	86.2	[S2]
0.94(0.75$Bi_{0.5}Na_{0.5}TiO_3$-0.25$Bi_{0.5}K_{0.5}TiO_3$)-0.06$BiAlO_3$	105	1.15	73.2	[S3]
0.76$Bi_{0.5}Na_{0.5}TiO_3$-0.24$SrTiO_3$-0.05$AgNbO_3$	120	2.03	61.8	[S4]
($Na_{0.775}K_{0.2}Li_{0.025}$)$_{0.5}Bi_{0.5}Ti_{0.985}Ta_{0.015}O_3$	130	1.29	64.5	[S5]
$Bi_{0.41}Na_{0.35}Sr_{0.21}TiO_3$	135	2.04	82.4	[S6]
0.8(0.98(0.94$Bi_{0.5}Na_{0.5}TiO_3$-0.06$BaTiO_3$)−0.02$Sr_{0.8}Na_{0.4}Nb_2O_6$)-0.2$NaNbO_3$	140	1.52	82	[S7]
{$Bi_{0.5}$[($Na_{0.8}K_{0.2}$)$_{0.9}Li_{0.1}$]$_{0.5}$}$_{0.96}Sr_{0.04}$($Ti_{0.975}Ta_{0.025}$)O_3	143	2.42	64	[S8]
0.7$Na_{0.46}Bi_{0.54}TiO_3$-0.3$BaSnO_3$	145.3	1.51	81.2	[S9]
0.9$Na_{0.5}Bi_{0.5}TiO_3$-0.1$BaZn_{1/3}Ta_{2/3}O_3$	150	1.07	71.5	[S10]
0.86{0.96($Bi_{0.5}Na_{0.5}$)($Ti_{0.995}Mn_{0.005}$)O_3-0.04$BiAlO_3$}-0.14$NaNbO_3$	153.8	2.01	71	[S11]
0.85$Na_{0.5}Bi_{0.5}TiO_3$-0.15$BaMg_{1/3}Nb_{2/3}O_3$	155	2.37	81.5	[S12]
($Bi_{1/2}Na_{1/2}$)$_{0.6}Sr_{0.4}Ti_{0.98}$($Fe_{1/2}Nb_{1/2}$)$_{0.02}O_3$	170	3.36	81	[S13]
0.85$Na_{0.5}Bi_{0.5}TiO_3$-0.15$BaHfO_3$	175	2.1	66.1	[S14]
0.85$Na_{0.5}Bi_{0.5}TiO_3$-0.15$BaMg_{1/3}Ta_{2/3}O_3$	175	2.8	78.67	[S15]
0.9(0.8$Bi_{0.5}Na_{0.5}TiO_3$-0.2$Ba_{0.3}Sr_{0.7}TiO_3$)-0.1$NaNbO_3$	180	2.26	87.34	[S16]

$(0.5Na_{0.5}Bi_{0.5}TiO_3\text{-}0.5SrTiO_3)$ -1%SnO_2	180	2.35	80	[S17]
$0.45(Bi_{0.5}Na_{0.5})TiO_3\text{-}0.45(BaZr_{0.3}Ti_{0.7}O_3)$	180	2.62	94	[S18]
$0.94(0.65Bi_{0.5}Na_{0.5}TiO_3\text{–}0.35Bi_{0.1}Sr_{0.85}TiO_3)\text{–}0.06K_{0.5}Na_{0.5}NbO_3$	180	2.65	84.6	[S19]
$0.825(Bi_{0.5}Na_{0.5})TiO_3\text{-}0.05BaTiO_3\text{-}0.125Bi(Zn_{2/3}Nb_{1/3})O_3$	180	2.83	67	[S20]
$0.7Bi_{0.5}Na_{0.5}TiO_3\text{-}0.3Sr_{0.7}Nd_{0.2}TiO_3$	182	3.09	77	[S21]
$0.85(Bi_{0.47}La_{0.03}Na_{0.5})_{0.94}Ba_{0.06}TiO_3\text{-}0.15Sr(Sc_{0.5}Nb_{0.5})O_3$	185	1.83	82.32	[S22]
$0.6Bi_{0.5}Na_{0.5}TiO_3\text{-}0.4(Sr_{0.775}Bi_{0.15})TiO_3$	190	2.41	89.5	[S23]
$0.75Bi_{0.5}Na_{0.5}TiO_3\text{-}0.25BaSnO_3$	190	19.1	86.4	[S24]
$(Na_{0.5}Bi_{0.5})_{0.8}Ba_{0.2}Ti_{0.8}Sn_{0.8}O_3$	195	2.35	71.04	[S25]
$0.75Na_{0.25}Sr_{0.5}Bi_{0.25}TiO_3\text{-}0.25MgO$	200	2.06	84	[S26]
$0.9Na_{0.5}Bi_{0.5}TiO_3\text{-}0.1Li_2TiO_3$	200	2.3	74.2	[S27]
$0.55Na_{0.5}Bi_{0.5}TiO_3\text{-}0.45(Bi_{0.2}Sr_{0.7}TiO_3)$	200	2.5	95	[S28]
$Na_{0.5}Bi_{0.5}TiO_3\text{-}0.3NaNbO_3$	200	2.64	90	[S29]
$0.7\ [0.85BaTiO_3\text{-}0.15Bi(Mg_{2/3}Nb_{1/3})O_3]\text{-}0.3Na_{0.5}Bi_{0.5}TiO_3$	200	2.91	85.55	[S30]
$0.9[0.76Bi_{0.5}Na_{0.5}TiO_3\text{-}0.24NaNbO_3]\text{-}0.1Sr_{0.7}Bi_{0.2}TiO_3$	200	3.12	75.3	[S31]
$0.6[0.935(Bi_{0.5}Na_{0.5})TiO_3\text{–}0.065BaTiO_3]\text{–}0.4Ba(Zr_{0.3}Ti_{0.7})O_3$	210	1.9	86	[S32]
$0.7[0.8Bi_{0.5}Na_{0.5}TiO_3\text{-}0.2Ba(Zr_{0.3}Ti_{0.7})O_3]\text{-}0.3Sr_{0.7}La_{0.2}TiO_3$	210	2.6	92.2	[S33]
$0.4Bi_{0.5}Na_{0.5}TiO_3\text{-}0.6Sr_{0.7}Ca_{0.3}TiO_3$-0.2 wt%$MnO_2$	210	2.64	70	[S34]
$[Ca_{0.1}(Bi_{0.5}Na_{0.5})_{0.9}](Ti_{0.85}Zr_{0.15})O_3$	210	2.79	76	[S35]
$0.94Bi_{0.5}Na_{0.5}TiO_3$- $0.06BaTiO_3$	210	3	75	[S36]
$0.97Na_{0.5}Bi_{0.5}TiO_3\text{-}0.03LaAlO_3$	210	3.18	60	[S37]
$0.775\ Bi_{0.5}Na_{0.5}TiO_3\text{-}0.225\ BaSnO_3$-5 wt% MgO	215	2.13	67.8	[S38]
$0.97[0.5(Bi_{0.5}Na_{0.5})TiO_3\text{-}0.5SrTiO_3]\text{-}0.03NaNbO_3$	220	2.34	74.6	[S39]
$0.96(0.65(Bi_{0.5}Na_{0.5})TiO_3\text{-}0.35Sr_{0.85}Bi_{0.1}TiO_3)\text{-}0.04NaNbO_3$	220	3.08	81.4	[S40]
$0.5(Bi_{0.5}Na_{0.5})TiO_3\text{-}0.5SrTiO_3$-1.5 mol% CuO	230	2.2	72.39	[S41]
$0.85(0.95Bi_{0.5}Na_{0.5}TiO_3\text{–}0.05SrZrO_3)\text{-}0.15NaNbO_3$	230	3.14	79	[S42]
$0.93(0.45SrTiO_3\text{–}0.2Na_{0.5}Bi_{0.5}TiO_3\text{–}0.35BaTiO_3)\text{–}0.07SnO_2$	240	2.25	79.51	[S43]
$0.84Bi_{0.5}Na_{0.5}TiO_3\text{-}0.16(Na_{0.73}Bi_{0.09})NbO_3$	240	2.41	81.6	[S44]
$0.9(0.7\ Bi_{0.5}Na_{0.5}TiO_3\text{-}0.3SrTiO_3)\text{-}0.1\ Bi(Nb_{0.5}Mg_{0.5})O_3$	240	3.46	78	[S45]
$0.8(0.775Na_{0.5}Bi_{0.5}TiO_3\text{-}0.225BaSnO_3)\text{-}0.2BaZrO_3$	245	2.08	88.8	[S46]
$0.95(0.6Bi_{0.5}Na_{0.5}TiO_3\text{–}0.4Sr_{0.7}Bi_{0.2}TiO_3)\text{-}0.05\ AgNbO_3$	246	3.62	89	[S47]
$0.3Bi_{0.5}Na_{0.5}TiO_3\text{-}0.7Sr_{0.85}Bi_{0.1}TiO_3$	250	2.32	80.1	[S48]
$0.85(Na_{0.5}Bi_{0.5})_{0.7}Sr_{0.3}TiO_3\text{–}0.15Bi(Mg_{2/3}Nb_{1/3})O_3$	250	3.45	88.01	[S49]

$0.98(Bi_{0.5}Na_{0.5}TiO_3–SrTiO_3)-0.02BaBi_2Nb_2O_9$	250	3.97	81	[S50]
$0.76Bi_{0.5}Na_{0.5}TiO_3-0.24SrTiO_3-Bi(Ni_{2/3}Nb_{1/3})O_3$	250	4.1	83.15	[S51]
$0.6\ Bi_{0.5}Na_{0.5}TiO_3-0.4Sr_{0.7}Sm_{0.2}TiO_3$	260	3.52	84.2	[S52]
$0.6(0.8Bi_{0.5}Na_{0.4}K_{0.1}TiO_3-0.2SrTiO_3)-0.4NaNbO_3$	260	4.44	81.8	[S53]
$0.90(Na_{0.5}Bi_{0.5})_{0.7}Sr_{0.3}TiO_3-0.10Bi(Ni_{0.5}Sn_{0.5})O_3$	270	4.18	83.64	[S54]
$0.55\ Bi_{0.5}Na_{0.5}TiO_3-0.45(Sr_{0.7}La_{0.2})(Ti_{0.8}Sn_{0.2})O_3$	280	2.52	89.3	[S55]
$0.6(Bi_{0.51}Na_{0.47})TiO_3-0.4Ba(Zr_{0.3}Ti_{0.7})O_3$	280	3.1	91	[S56]
$0.94(0.65\ Bi_{0.5}Na_{0.5}TiO_3-0.35Sr_{0.7}Bi_{0.2}TiO_3)-0.06K_{0.5}Na_{0.5}NbO_3$	280	3.25	93.8	[S57]
$0.88Bi_{0.47}Na_{0.47}Ba_{0.06}TiO_3-0.12CaHfO_3$	280	4.2	66.7	[S58]
99.5 mol%[$0.6SrTiO_3-0.4Na_{0.5}Bi_{0.5}TiO_3$]-0.5 mol%$ZrO_2$	285	2.84	71.54	[S59]
$0.6(Ba_{0.7}Sr_{0.3})(Zr_{0.2}Ti_{0.8})O_3-0.4(Na_{0.5}Bi_{0.5})TiO_3$	289	3.72	94.3	[S60]
$0.85(0.75Na_{0.5}Bi_{0.5}TiO_3-0.25SrTiO_3)-0.15Ag(Nb_{0.85}Ta_{0.15})O_3$	290	3.6	80	[S61]
$0.85[0.7\ Na_{0.5}Bi_{0.5}TiO_3-0.3Bi_{0.1}Sr_{0.85}TiO_3]-0.15KNbO_3$	290	3.72	90.7	[S62]
$0.76(0.94Na_{0.5}Bi_{0.5}TiO_3-0.06BaTiO_3)-0.24(Sr_{0.85}Bi_{0.1})(Mg_{1/3}Nb_{2/3})O_3$	300	3.05	89	[S63]
$0.5\ Na_{0.5}Bi_{0.5}TiO_3-0.5Sr_{0.7}Nd_{0.2}TiO_3$	305	3.85	85.3	[S64]
$(Na_{0.25}Bi_{0.25}Sr_{0.5})(Ti_{0.8}Sn_{0.2})O_3$	310	3.4	90	[S65]
$0.55Bi_{0.5}Na0._{5}TiO_3-0.45Sr_{0.7}La_{0.2}TiO_3$	315	4.14	92.2	[S66]
$0.9(0.65\ Bi_{0.5}Na0._{5}TiO_3-0.35Bi_{0.2}Sr_{0.7}TiO_3)-0.1CaZrO_3$	330	2.9	80	[S67]
$0.85(0.55Na_{0.5}Bi_{0.5}TiO_3-0.45Sr_{0.7}La_{0.2}TiO_3)-0.15Bi(Mg_{2/3}Nb_{1/3})O_3$	338	3.88	85	[S68]
$0.65(0.84Bi_{0.5}Na_{0.5}TiO_3–0.16K_{0.5}Bi_{0.5}TiO_3)–0.35(Bi_{0.2}Sr_{0.7}TiO_3)$	350	4.06	87.3	[S69]
$0.91Bi_{0.5}Na_{0.5}TiO_3-0.09(0.5Ba_{0.7}Ca_{0.3}TiO_3-0.5BaTi_{0.8}Zr_{0.2}O_3)$	360	3.49	64.9	[S70]
$0.88Na_{0.5}Bi_{0.5}TiO_3-0.12CaZr_{0.5}Ti_{0.5}O_3$	378.3	4.77	69	[S71]
$0.8(0.65Bi_{0.5}Na_{0.5}TiO_3-0.35Bi_{0.2}Sr_{0.7}TiO_3)-0.2BaSnO_3$	380	3.75	84.8	[S72]
$0.8Bi_{0.5}Na_{0.5}TiO_3–0.2NaTaO_3$	380	4.21	77.8	[S73]
$0.95(0.9Bi_{0.5}Na_{0.5}TiO_3-0.1Ba_{0.85}Ca_{0.15}Zr_{0.1}Ti_{0.9}O_3)-0.05BaSnO_3$	385	4.97	84.4	[S74]
$0.85(0.94Bi_{0.5}Na_{0.5}TiO_3-0.06BaTiO_3)-0.15BiMg_{2/3}Nb_{1/3}O_3$	420	6.3	80	[S75]
$0.5Na_{0.5}Bi_{0.5}TiO_3-0.5Sr_{0.85}Sm_{0.1}TiO_3$	422	5.02	90	[S76]
$(Na_{0.5}Bi_{0.5})_{0.7}Sr_{0.3}TiO_3-Ba(Mg_{1/3}Nb_{2/3})O_3$	460	5.5	90.1	[S77]
$0.8Bi_{0.5}Na_{0.5}TiO_3-0.2SrNb_{0.5}Al_{0.5}O_3$	520	6.64	96	[S78]
$0.75Bi_{(0.58)}Na_{(0.42)}TiO_3-0.25SrTiO_3$	535	5.63	94	[S79]

$0.94Bi_{0.55}Na_{0.45}TiO_3$–$0.06BaTiO_3$-$0.80Bi_{0.5}Na_{0.5}TiO_3$–$0.20SrNb_{0.5}Al_{0.5}O_3$	540	6.3	93.61	[S80]
$0.7(0.85Bi_{0.5}Na_{0.5}TiO_3$–$0.15NaNbO_3)$-$0.3(Sr_{1.05}Bi_{0.3})ScO_3$	540	7.3	80	[S81]
$(Bi_{0.5}(Na_{0.8}K_{0.2})_{0.5})_{0.96}Sr_{0.04}Ti_{0.99}Ta_{0.01}O_3$-$0.70Bi_{0.5}Na_{0.5}TiO_3$-$0.30SrNb_{0.5}Al_{0.5}O_3$	572	6.78	89.7	[S82]

Table S2 Characterized and crystal structure fitting parameters and results for (1-x)$Bi_{0.5}Na_{0.1}K_{0.1}TiO_3$-$x$($1/3SrTiO_3$-$2/3Bi(Mg_{2/3}Nb_{1/3})O_3$) ceramics

		x=0.35	x=0.40	x=0.45	x=0.50
AGS (μm)		1.60	1.68	1.61	1.66
Density (g/cm^3)		5.62	5.65	5.68	5.66
Crystal system		R+T	R+T	R+T	R+T
Space group		*R*3*c*+*P*4*bm*	*R*3*c*+*P*4*bm*	*R*3*c*+*P*4*bm*	*R*3*c*+*P*4*bm*
Cell parameter (Å)	R	*a*=5.5450(6)	*a*=5.5391(9)	*a*=5.5525(4)	*a*=5.5585(1)
		b=5.5450(6)	*b*=5.5391(9)	*b*=5.5525(4)	*b*=5.5585(1)
		c=13.5734(4)	*c*=13.5066(9)	*c*=13.5520(0)	*c*=13.6070(6)
	T	*a*=5.5458(5)	*a*=5.5470(0)	*a*=5.5533(9)	*a*=5.5586(0)
		b=5.5458(5)	*b*=5.5470(0)	*b*=5.5533(9)	*b*=5.5586(0)
		c=3.9207(8)	*c*=3.9204(2)	*c*=3.9287(4)	*c*=3.9308(9)
Volume (Å^3)	R	361.44	358.90	361.84	364.09
	T	120.59	120.63	121.15	121.46
Fraction (%)	R	61.76	68.33	70.51	69.13
	T	38.24	31.67	29.49	30.87
R-factor [a]	R_p (%)	6.75	7.33	6.78	6.57
	R_{wp} (%)	9.87	9.56	9.78	9.76
	χ^2 (%)	1.56	1.42	1.60	1.54
No. of profile points used		12001	12001	12001	12001

[a] For the definition of *R*-factor see Ref. [83].

Table S3 Energy storage properties of this work compared with other BNT-based bulk ceramics

Ceramic compositions	E_{max} (kV/cm)	W_{rec} (J/cm^3)	η (%)	Refs.
$0.95(0.6Bi_{0.5}Na_{0.5}TiO_3$-$0.4Sr_{0.7}Bi_{0.2}TiO_3)$-$0.05AgNbO_3$	246	3.62	89	[S84]
$0.75Bi_{0.58}Na_{0.42}TiO_3$-$0.25SrTiO_3$	569	5.63	94	[S79]
$0.76(Bi_{0.5}Na_{0.5})TiO_3$-$0.24NaNbO_3$	200	3.12	75	[S31]
$0.90(Na_{0.5}Bi_{0.5})_{0.7}Sr_{0.3}TiO_3$-$0.10Bi(Ni_{0.5}Sn_{0.5})O_3$	270	4.18	83	[S54]
$(Na_{0.25}Bi_{0.25}Sr_{0.5})(Ti_{0.8}Sn_{0.2})O_3$	310	3.4	90	[S85]
$0.78(Bi_{0.5}Na_{0.5})TiO_3$-$0.22NaNbO_3$	390	7.02	85	[S86]
$0.8SrTiO_3$-$0.2(0.93Bi_{0.5}Na_{0.5}TiO_3$-$0.07Ba_{0.94}La_{0.04}Zr_{0.02}Ti_{0.98}O_3)$	250	2.79	76	[S87]
$[Ca_{0.05}(Bi_{0.5}Na_{0.5})_{0.95}](Ti_{0.85}Zr_{0.15})O_3$	200	3.12	75	[S35]
$Na_{0.25}Sr_{0.5}Bi_{0.25}TiO_3$-$0.25MgO$	170	3.36	81	[S26]
$(Bi_{1/2}Na_{1/2})_{0.6}Sr_{0.4}Ti_{0.98}(Fe_{1/2}Nb_{1/2})_{0.02}O_3$	190	1.67	80	[S13]
$Na_{0.3}Sr_{0.4}Bi_{0.3}TiO_3$-$0.02La$	210	2.15	81	[S88]
$0.85Na_{0.5}Bi_{0.5}TiO_3$-$0.15CaTiO_3$-$0.06La$	280	4.2	66	[S89]
$0.88Bi_{0.47}Na_{0.47}Ba_{0.06}TiO_3$-$0.12CaHfO_3$	290	3.6	80	[S58]
$0.85(0.75Na_{0.5}Bi_{0.5}TiO_3$-$0.25SrTiO_3)$-$0.15Ag(Nb_{0.85}Ta_{0.15})O_3$	270	3.12	88	[S90]
$0.9(Na_{0.4}Bi_{0.4}Ba_{0.06}Sr_{0.14}Ti_{0.99}Ta_{0.01}O_3)$-$0.1NaNbO_3$	290	3.72	91	[S91]
$0.85[0.7Bi_{0.5}Na_{0.5}TiO_3$-$0.3Bi_{0.1}Sr_{0.85}TiO_3]$-$0.15KNbO_3$	569	5.63	94	[S62]
$0.75Bi_{0.4465}Na_{0.4465}Ba_{0.057}La_{0.05}TiO_3$-$0.25Sr_{0.85}Bi_{0.1}TiO_3$	320	4.55	90	[S92]
$0.55Bi_{0.5}Na_{0.5}TiO_3$-$0.45Sr_{0.7}La_{0.2}TiO_3$	338	4.14	92	[S66]
$(1\text{-}x)Bi_{0.5}Na_{0.5}TiO_3$-$xSrNb_{0.5}Al_{0.5}O_3$	520	6.64	96	[S78]
$0.85(Na_{0.5}Bi_{0.5})_{0.7}Sr_{0.3}TiO_3$-$0.15Bi(Mg_{2/3}Nb_{1/3})O_3$	250	3.45	88	[S93]
$0.85(0.94Bi_{0.5}Na_{0.5}TiO_3$-$0.06BaTiO_3)$-$0.15BiMg_{2/3}Nb_{1/3}O_3$	420	6.3	80	[S75]
$0.5Bi_{0.5}Na_{0.4}K_{0.1}TiO_3$-$0.5(2/3SrTiO_3$-$1/3Bi(Mg_{2/3}Ni_{1/3})O_3)$	460	7.19	93.8	This work

Table S4 Energy storage properties of this work compared with other representative bulk ceramics

Ceramic compositions	E_{max} (kV/cm)	W_{rec} (J/cm^3)	η (%)	Refs.
$0.85Na_{0.35}Bi_{0.35}Sr_{0.3}TiO_3$–$0.15BiMg_{2/3}Nb_{1/3}O_3$	250	3.45	88.01	[S94]
$0.98(Bi_{0.5}Na_{0.5}TiO_3$–$SrTiO_3)$-$0.02BaBi_2Nb_2O_9$	250	3.97	81	[S50]
$0.76Bi_{0.5}Na_{0.5}TiO_3$-$0.24SrTiO_3$-$Bi(Ni_{2/3}Nb_{1/3})O_3$	250	4.1	83.15	[S51]
0.6 $Bi_{0.5}Na_{0.5}TiO_3$-$0.4Sr_{0.7}Sm_{0.2}TiO_3$	260	3.52	84.2	[S52]
$0.6(0.8Bi_{0.5}Na_{0.4}K_{0.1}TiO_3$-$0.2SrTiO_3)$-$0.4NaNbO_3$	260	4.44	81.8	[S53]
$0.90(Na_{0.5}Bi_{0.5})_{0.7}Sr_{0.3}TiO_3$-$0.10Bi(Ni_{0.5}Sn_{0.5})O_3$	270	4.18	83.64	[S54]
0.55 $Bi_{0.5}Na_{0.5}TiO_3$-$0.45(Sr_{0.7}La_{0.2})(Ti_{0.8}Sn_{0.2})O_3$	280	2.52	89.3	[S55]
$0.6(Bi_{0.51}Na_{0.47})TiO_3$-$0.4Ba(Zr_{0.3}Ti_{0.7})O_3$	280	3.1	91	[S56]
0.94(0.65 $Bi_{0.5}Na_{0.5}TiO_3$-$0.35Sr_{0.7}Bi_{0.2}TiO_3)$-$0.06K_{0.5}Na_{0.5}NbO_3$	280	3.25	93.8	[S57]
$0.88Bi_{0.47}Na_{0.47}Ba_{0.06}TiO_3$-$0.12CaHfO_3$	280	4.2	66.7	[S58]
99.5 mol%[$0.6SrTiO_3$-$0.4Na_{0.5}Bi_{0.5}TiO_3$]-0.5 mol%ZrO_2	285	2.84	71.54	[S59]
$0.6(Ba_{0.7}Sr_{0.3})(Zr_{0.2}Ti_{0.8})O_3$-$0.4(Na_{0.5}Bi_{0.5})TiO_3$	289	3.72	94.3	[S60]
$0.85(0.75Na_{0.5}Bi_{0.5}TiO_3$-$0.25SrTiO_3)$-$0.15Ag(Nb_{0.85}Ta_{0.15})O_3$	290	3.6	80	[S61]
0.85[0.7 $Na_{0.5}Bi_{0.5}TiO_3$-$0.3Bi_{0.1}Sr_{0.85}TiO_3$]-$0.15KNbO_3$	290	3.72	90.7	[S62]
$0.76(0.94Na_{0.5}Bi_{0.5}TiO_3$-$0.06BaTiO_3)$-$0.24(Sr_{0.85}Bi_{0.1})(Mg_{1/3}Nb_{2/3})O_3$	300	3.05	89	[S63]
0.5 $Na_{0.5}Bi_{0.5}TiO_3$-$0.5Sr_{0.7}Nd_{0.2}TiO_3$	305	3.85	85.3	[S64]
$(Na_{0.25}Bi_{0.25}Sr_{0.5})(Ti_{0.8}Sn_{0.2})O_3$	310	3.4	90	[S65]
$0.55Bi_{0.5}Na0_{.5}TiO_3$-$0.45Sr_{0.7}La_{0.2}TiO_3$	315	4.14	92.2	[S66]
0.9(0.65 $Bi_{0.5}Na0_{.5}TiO_3$-$0.35Bi_{0.2}Sr_{0.7}TiO_3)$-$0.1CaZrO_3$	330	2.9	80	[S67]
$0.85(0.55Na_{0.5}Bi_{0.5}TiO_3$-$0.45Sr_{0.7}La_{0.2}TiO_3)$-$0.15Bi(Mg_{2/3}Nb_{1/3})O_3$	338	3.88	85	[S68]
$0.65(0.84Bi_{0.5}Na_{0.5}TiO_3$–$0.16K_{0.5}Bi_{0.5}TiO_3)$–$0.35(Bi_{0.2}Sr_{0.7}TiO_3)$	350	4.06	87.3	[S69]
$0.91Bi_{0.5}Na_{0.5}TiO_3$-$0.09(0.5Ba_{0.7}Ca_{0.3}TiO_3$-$0.5BaTi_{0.8}Zr_{0.2}O_3)$	360	3.49	64.9	[S70]
$0.88Na_{0.5}Bi_{0.5}TiO_3$-$0.12CaZr_{0.5}Ti_{0.5}O_3$	378.3	4.77	69	[S71]
$0.8(0.65Bi_{0.5}Na_{0.5}TiO_3$-$0.35Bi_{0.2}Sr_{0.7}TiO_3)$-$0.2BaSnO_3$	380	3.75	84.8	[S72]
$0.8Bi_{0.5}Na_{0.5}TiO_3$–$0.2NaTaO_3$	380	4.21	77.8	[S73]
$0.95(0.9Bi_{0.5}Na_{0.5}TiO_3$-$0.1Ba_{0.85}Ca_{0.15}Zr_{0.1}Ti_{0.9}O_3)$-$0.05BaSnO_3$	385	4.97	84.4	[S74]
$0.85(0.94Bi_{0.5}Na_{0.5}TiO_3$-$0.06BaTiO_3)$-$0.15BiMg_{2/3}Nb_{1/3}O_3$	420	6.3	80	[S75]
$0.5Na_{0.5}Bi_{0.5}TiO_3$-$0.5Sr_{0.85}Sm_{0.1}TiO_3$	422	5.02	90	[S76]
$(Na_{0.5}Bi_{0.5})_{0.7}Sr_{0.3}TiO_3$-$Ba(Mg_{1/3}Nb_{2/3})O_3$	460	5.5	90.1	[S77]
$0.8Bi_{0.5}Na_{0.5}TiO_3$-$0.2SrNb_{0.5}Al_{0.5}O_3$	520	6.64	96	[S78]

$0.75Bi_{(0.58)}Na_{(0.42)}TiO_3$-$0.25SrTiO_3$	535	5.63	94	[S79]
$0.94Bi_{0.55}Na_{0.45}TiO_3$–$0.06BaTiO_3$-$0.80Bi_{0.5}Na_{0.5}TiO_3$–$0.20SrNb_{0.5}Al_{0.5}O_3$	540	6.3	93.61	[S80]
$(Bi_{0.5}(Na_{0.8}K_{0.2})_{0.5})_{0.96}Sr_{0.04}Ti_{0.99}Ta_{0.01}O_3$- $0.70Bi_{0.5}Na_{0.5}TiO_3$-$0.30SrNb_{0.5}Al_{0.5}O_3$	572	6.78	89.7	[S82]
$[0.94(Bi_{0.5}Na_{0.5})TiO_3$-$0.06BaTiO_3]$-$0.02Ba(Sn_{0.70}Nb_{0.24})O_3$	100	1.28	74	[S1]
$0.7Na_{0.5}Bi_{0.5}TiO_3$-$0.3Sr_{0.7}Bi_{0.2}TiO_3$-0.05Mn	100	1.33	86.2	[S2]
$0.94(0.75Bi_{0.5}Na_{0.5}TiO_3$-$0.25Bi_{0.5}K_{0.5}TiO_3)$-$0.06BiAlO_3$	105	1.15	73.2	[S3]
$0.76Bi_{0.5}Na_{0.5}TiO_3$-$0.24SrTiO_3$-$0.05AgNbO_3$	120	2.03	61.8	[4S]
$(Na_{0.775}K_{0.2}Li_{0.025})_{0.5}Bi_{0.5}Ti_{0.985}Ta_{0.015}O_3$	130	1.29	64.5	[S5]
$Bi_{0.41}Na_{0.35}Sr_{0.21}TiO_3$	135	2.04	82.4	[S6]
0.91NaNbO3-0.09Bi(Zn0.5Ti0.5)O3	200	2.1	76	[S95]
$NaNbO_3$–$Bi(Mg_{2/3}Nb_{1/3})NbO_3$	300	2.8	82	[S96]
0.80 $NaNbO_3$-0.20 $SrTiO_3$	323	3.02	80.7	[S97]
$0.78NaNbO_3$-$0.22Ba(Mg_{1/3}Nb_{2/3})O_3$	540	3.51	87	[S98]
$Na_{0.7}Bi_{0.1}NbO_3$	250	4.03	85	[S99]
$NaNbO_3$-MnO	360	4.3	90	[S100]
$(Na_{0.84}Bi_{0.08})(Nb_{0.92}Zr_{0.08})O_3$	430	4.9	88	[S101]
$0.9NaNbO_3$-$0.1Bi(Ni_{1/2}Sn_{1/2})O_3$	550	5	68	[S102]
$0.78NaNbO_3$–$0.22Bi(Mg_{2/3}Ta_{1/3})O_3$	620	5.01	86.8	[S103]
$NaNbO_3$–$Bi(Mg_{0.5}Ti_{0.5})O_3$	480	5.57	71	[S104]
$(Na_{0.91}La_{0.09})(Nb_{0.82}Ti_{0.18})O_3$	550	6.5	65.9	[S105]
$0.75[0.90NaNbO_3$–$0.10Bi(Mg_{0.5}Ta_{0.5})O_3]$–$0.25(Bi_{0.5}Na_{0.5})_{0.7}Sr_{0.3}TiO_3$	800	8	90	[S106]
0.50 $NaNbO_3$-0.50 $NaTaO_3$	300	2.2	80.10%	[S107]
0.7 $NaNbO_3$-0.3 $CaTiO_3$	560	5.52	83.3	[S108]
$0.89NaNbO_3$-$0.11Bi_{0.5}K_{0.5}ZrO_3$	400	4.4	90	[S109]
$0.85BaTiO_3$–$0.15Bi(Mg_{2/3}Nb_{1/3})O_3$	143.5	1.13	95	[S110]
$0.95Ba_{0.85}Ca_{0.15}Zr_{0.1}Ti_{0.9}O_3$-$0.05(B_2O_3$-$Al_2O_3$-$SiO_2)$	200	1.153	/	[S111]
$0.9BaTiO_3$-$0.1(Bi_{0.9}Na_{0.1})(In_{0.8}Zr_{0.2})O_3$	180	1.33	88	[S112]
$0.88(Ba_{0.8}Sr_{0.2})TiO_3$-$0.12Bi(Zn_{2/3}Nb_{1/3})O_3$	225	1.62	99	[S113]
$0.9BaTiO_3$-$0.1Bi(Mg_{2/3}Nb_{1/3})O_3$	205	1.7	88	[S114]
$0.92(0.65BaTiO_3$-$0.35Bi_{0.5}Na_{0.5}TiO_3)$-$0.08Na_{0.73}Bi_{0.09}NbO_3$	172	1.7	82	[S115]
$0.92(0.92BaTiO_3$–$0.08K_{0.5}Bi_{0.5}TiO_3)$–$0.08NaNbO_3$	220	1.96	67	[S116]
$0.88BaTiO_3$–$0.12Bi(Li_{0.5}Nb_{0.5})O_3$	270	2.03	88	[S117]
$0.9BaTiO_3$–$0.1Bi(Li_{0.5}Ta_{0.5})O_3$	280	2.2	88	[S118]

$0.9BaTiO_3$–$0.1Bi(Zn_{0.5}Zr_{0.5})O_3$	264	2.46	/	[S119]
$BaTiO_3$-$0.06Bi_{2/3}(Mg_{1/3}Nb_{2/3})O_3$	520	4.55	91	[S120]
$0.6BaTiO_3$-$0.4Bi(Mg_{1/2}Ti_{1/2})O_3$	340	4.49	93	[S121]
$0.9BaTiO_3$-$0.1(Sr_{0.7}Bi_{0.2})TiO_3$	410	3.5	77	[S122]
$0.9Ba_{0.65}Sr_{0.35}TiO_3$–$0.1Bi(Mg_{2/3}Nb_{1/3})O_3$	400	3.34	85.7	[S123]
$0.85(0.8BaTiO_3$-$0.2Bi_{0.5}Na_{0.5}TiO_3)$-$0.15CaZrO_3$	540	9.04	87.2	[S124]
$0.95(0.65BiFeO_3$-$0.35BaTiO_3)$-$0.05Nb_2O_5$	90	0.71	/	[S125]
$0.75(Bi_{0.9}Nd_{0.1})FeO_3$-$0.25BaTiO_3$	170	1.82	41	[S126]
$0.57BiFeO_3$–$0.3BaTiO_3$–$0.13Bi(Li_{0.5}Nb_{0.5})O_3$	280	3.64	74	[S127]
$0.61BiFeO_3$-$0.33(Ba_{0.8}Sr_{0.2})TiO_3$-$0.06La(Mg_{2/3}Nb_{1/3})O_3$+0.1 wt.% MnO_2+2 wt.% $BaCu(B_2O_5)$	230	3.38	59	[S128]
$0.25Bi_{0.83}Sm_{0.17}Fe_{0.95}Sc_{0.05}O_3$-$0.75(0.85BaTiO_3$-$0.15Bi(Mg_{0.5}Zr_{0.5})O_3)$	206	3.2	92	[S129]
$0.6BiFeO_3$–$0.34BaTiO_3$–$006Ba(Zn_{1/3}Ta_{2/3})O_3$	160	2.56	71	[S130]
$0.62BiFeO_3$–$0.3BaTiO_3$–0.08Nd	240	2.45	54	[S131]
$0.35BiFeO_3$-$0.65SrTiO_3$	626	7.5	90	[S132]
$0.46Bi_{1.02}FeO_3$-$0.29BaTiO_3$-$0.25Bi_{0.5}Na_{0.5}$TiO3-1%Nb_2O_5	250	3.9	80	[S133]
$0.7(Bi_{0.95}Nd_{0.05})[SFe_{0.95}(Li_{0.5}Nb_{0.5})_{0.05}]O_3$-$0.3BaTiO_3$+0.1 wt% MnO_2	290	3.2	73.7	[S134]
95 wt%$Ba_{0.4}Sr_{0.6}TiO_3$–5 wt%MgO	300	1.5	88.5	[S135]
$(Ba,Sr)_2TiSi_2O_8$	400	1.6	90.9	[S136]
$0.6SrTiO_3$-$0.4(Na_{0.5}Bi_{0.5})TiO_3$	210	1.7	70	[S137]
$0.45SrTiO_3$-$0.2Na_{0.5}Bi_{0.5}TiO_3$-$0.35BaTiO_3$	170	1.78	77	[S138]
$0.99SrTiO_3$-0.01MgO	362	1.86	89.3	[S139]
$Ba_{0.4}Sr_{0.6}(Ti_{0.996}Mn_{0.004})O_3$–2 wt% MgO	300	2.01	88.6	[S140]
$Sr_{0.985}Ce_{0.01}TiO_3$ @3 wt% SiO_2	290	2.23	/	[S141]
$SrTi_{0.985}(Zn_{1/3}Nb_{2/3})_{0.015}O_3$–4.5 wt%$ZnNb_2O_6$	422	2.35	77	[S142]
$0.6SrTiO_3$-$0.4(Bi_{0.48}La_{0.02}Na_{0.48}Li_{0.02}Ti_{0.98}Zr_{0.02}O_3)$	323	2.59	85	[S143]
[S$0.6SrTiO_3$-$0.4Na_{0.5}Bi_{0.5}TiO_3$]-0.5 mol%ZrO_2	285	2.84	71	[S144]
$0.9(Sr_{0.7}Bi_{0.2})TiO_3$–$0.1Bi(Mg_{0.5}Hf_{0.5})O_3$	360	3.1	93	[S145]
$Ba_{0.3}Sr_{0.7}TiO_3$-1.6 wt.%SiO_2	400	3.9	/	[S146]
$Ba_{0.4}Sr_{0.6}TiO_3$-$3Al_2O_3$-SiO_2	493	5.09	/	[S147]
$(K_{0.48}Na_{0.52})_{0.88}$ Bi $_{0.04}$ NbO_3	189	1.04	/	[S148]
$0.93K_{0.5}Na_{0.5}NbO_3$–$0.07Bi_{0.6}(Mg_{1/3}Nb_{2/3})O_3$	150	1.3	58.8	[S149]
$0.9K_{0.5}Na_{0.5}NbO_3$-$0.1BiFeO_3$	206	2	61	[S150]
$0.8(K_{0.5}Na_{0.5})NbO_3$–$0.2Sr(Sc_{0.5}Nb_{0.5})O_3$	400	2.6	73.2	[S151]

$K_{0.14}Bi_{0.12}Na_{0.5}NbO_3$-1 mol%CuO	300	2.89	80	[S152]
$0.85K_{0.5}Na_{0.5}NbO_3$-$0.15Bi(Zn_{0.5}Zr_{0.5})O_3$	325	3.5	86.8	[S153]
$0.9(K_{0.5}Na_{0.5})NbO_3$–$0.1Bi(Mg_{2/3}Nb_{1/3})O_3$–1.0 mol% CuO	400	4.02	57.3	[S154]
$0.85(K_{0.5}Na_{0.5})NbO_3$–$0.15SrTiO_3$	400	4.03	52	[S155]
$0.9(K_{0.5}Na_{0.5})NbO_3$–$0.1Bi(Mg_{2/3}Nb_{1/3})O_3$	300	4.08	62.7	[S156]
$0.825(K_{0.5}Na_{0.5})NbO_3$-$0.175Sr(Sc_{0.5}Nb_{0.5})O_3$	395	4.42	60	[S157]
$Ag_{0.76}La_{0.08}NbO_3$	476	7.01	77	[S158]
$Ag_{0.97}Nd_{0.01}Ta_{0.20}Nb_{0.80}O_3$	370	6.5	71	[S159]
$0.45AgNbO_3$-$0.55AgTaO_3$	460	6.3	90	[S160]
$Sm_{0.03}Ag_{0.91}NbO_3$	290	5.2	69.2	[S161]
$(Sm_{0.02}Ag_{0.94})(Nb_{0.9}Ta_{0.1})O_3$	280	4.87	63.5	[S162]
$Ag_{0.91}Sm_{0.03}NbO_3$	310	4.5	63	[S163]
$(Ag_{0.90}Ca_{0.05})(Nb_{0.95}Ta_{0.05})O_3$	210	3.36	58	[S164]
$Ag_{0.97}La_{0.01}NbO_3$-0.3 wt % MnO	142	3.2	62	[S165]
$Ag_{0.9}Sr_{0.05}NbO_3$	190	2.9	56	[S166]
$0.94(Na_{0.50}Bi_{0.50})TiO_3 - 0.06KSbO_3$	150	2.5	57	[S167]
$AgNbO_3$	175	2.1	50	[S168]
$Ag_{0.94}Bi_{0.02}Nb_{0.988}Sc_{0.02}O_3$	215	3.65	84.31	[S169]
0.1 wt % MnO_2-$AgNbO_3$-SiO_2	300	5.9	71	[S170]
$0.5Bi_{0.5}Na_{0.4}K_{0.1}TiO_3$-$0.5(2/3SrTiO_3$-$1/3Bi(Mg_{2/3}Ni_{1/3})O_3)$	7.19	94	460	This work

Table S5 Temperature-dependent energy storage properties of this work compared with other BNT-based bulk ceramics

No.	Ceramic compositions	Refs.
1	$0.78(Bi_{0.5}Na_{0.5})TiO_3$-$0.22NaNbO_3$	[S86]
2	$0.8Bi_{0.5}Na_{0.5}TiO_3$-$0.2SrNb_{0.5}Al_{0.5}O_3$	[S78]
3	$0.75Bi_{0.58}Na_{0.42}TiO_3$-$0.25SrTiO_3$	[S79]
4	$0.75Bi_{0.4465}Na_{0.4465}Ba_{0.057}La_{0.05}TiO_3$-$0.25Sr_{0.85}Bi_{0.1}TiO_3$	[S92]
5	$0.90(Na_{0.5}Bi_{0.5})_{0.7}Sr_{0.3}TiO_3$-$0.10Bi(Ni_{0.5}Sn_{0.5})O_3$	[S54]
6	$0.94Bi_{0.50}(Na_{0.78}K_{0.22})_{0.50}Ti_{0.96}(Al_{0.50}Nb_{0.50})_{0.04}O_3$-$0.06BaZrO_3$	[S171]
7	$0.5Bi_{0.5}Na_{0.4}K_{0.1}TiO_3$-$0.5(2/3SrTiO_3$-$1/3Bi(Mg_{2/3}Ni_{1/3})O_3)$	This work

Supplementary References

[S1] Y. Jia, H. Fan, H. Wang, A.K. Yadav, B. Yan et al., Large electrostrain and high energy-storage of (1-x)[0.94($Bi_{0.5}Na_{0.5}$) TiO_3-0.06$BaTiO_3$]-xBa($Sn_{0.70}Nb_{0.24}$)O_3 lead-free ceramics. Ceram. Int. **47**, 18487–18496 (2021). https://doi.org/10.1016/j.ceramint.2021.03.172

[S2] J. Wang, H. Fan, Enhanced energy storage performance and fatigue resistance of Mn-doped 0.7$Na_{0.5}Bi_{0.5}$TiO3–0.3$Sr_{0.7}Bi_{0.2}TiO_3$ lead-free ferroelectric ceramics. J. Mater. Res. **36**, 1161–1170 (2021). https://doi.org/10.1557/s43578-020-00082-5

[S3] Z. Yu, Y. Liu, M. Shen, H. Qian, F. Li et al., Enhanced energy storage properties of $BiAlO_3$ modified $Bi_{0.5}Na_{0.5}TiO_3$–$Bi_{0.5}K_{0.5}TiO_3$ lead-free antiferroelectric ceramics. Ceram. Int. **43**, 7653–7659 (2017). https://doi.org/10.1016/j.ceramint.2017.03.062

[S4] W. Ma, Y. Zhu, M. Ali Marwat, P. Fan, B. Xie et al., Enhanced energy-storage performance with excellent stability under low electric fields in BNT–ST relaxor ferroelectric ceramics. J. Mater. Chem. C **7**, 281–288 (2019). https://doi.org/10.1039/c8tc04447c

[S5] H. Wang, Q. Li, Y. Jia, A.K. Yadav, B. Yan et al., $(Na_{0.8\text{-}x}K_{0.2}Li_x)_{0.5}Bi_{0.5}Ti_{0.985}Ta_{0.015}O_3$ lead-free ceramics with large strain and high recoverable energy density. J. Alloys Compd. **879**, 160378 (2021). https://doi.org/10.1016/j.jallcom.2021.160378

[S6] Z. Liu, A. Zhang, S. Xu, J. Lu, B. Xie et al., Mediating the confliction of polarizability and breakdown electric-field strength in BNST relaxor ferroelectric for energy storage applications. J. Alloys Compd. **823**, 153772 (2020). https://doi.org/10.1016/j.jallcom.2020.153772

[S7] Y. Wan, N. Hou, P. Ren, M. Ma, K. Song et al., High temperature energy storage properties of $Bi_{0.5}Na_{0.5}TiO_3$ based ceramics modified by $NaNbO_3$. J. Alloys Compd. **888**, 161591 (2021). https://doi.org/10.1016/j.jallcom.2021.161591

[S8] J. Yin, Y. Zhang, X. Lv, J. Wu, Ultrahigh energy-storage potential under low electric field in bismuth sodium titanate-based perovskite ferroelectrics. J. Mater. Chem. A **6**, 9823–9832 (2018). https://doi.org/10.1039/c8ta00474a

[S9] Z. Fan, Y. Yu, J. Huang, Q. Zhang, Y. Lu et al., Excellent energy storage properties over a wide temperature range under low driving electric fields in NBT-BSN lead-free relaxor ferroelectric ceramics. Ceram. Int. **47**, 4715–4721 (2021). https://doi.org/10.1016/j.ceramint.2020.10.040

[S10] M. Chen, Y. Pu, L. Zhang, Simultaneously achieving high energy-storage density and power density under low electric fields in NBT-based ceramics. Ceram. Int. **47**, 34521–34528 (2021). https://doi.org/10.1016/j.ceramint.2021.08.367

[S11] P. Peng, H. Nie, C. Zheng, G. Wang, X. Dong, High-energy storage density in

$NaNbO_3$-modified $(Bi_{0.5}Na_{0.5})TiO_3$-$BiAlO_3$-based lead-free ceramics under low electric field. J. Am. Ceram. Soc. **104**, 2610–2620 (2021). https://doi.org/10.1111/jace.17697

[S12] L. Zhang, Y. Pu, M. Chen, F. Zhuo, C. Dietz et al., Decreasing polar-structure size: achieving superior energy storage properties and temperature stability in $Na_{0.5}Bi_{0.5}TiO_3$-based ceramics for low electric field and high-temperature applications. J. Eur. Ceram. Soc. **41**, 5890–5899 (2021). https://doi.org/10.1016/j.jeurceramsoc.2021.05.036

[S13] R. Kang, Z. Wang, X. Lou, W. Liu, P. Shi et al., Energy storage performance of $Bi_{0.5}Na_{0.5}TiO_3$-based relaxor ferroelectric ceramics with superior temperature stability under low electric fields. Chem. Eng. J. **410**, 128376 (2021). https://doi.org/10.1016/j.cej.2020.128376

[S14] L. Zhang, Y. Pu, M. Chen, T. Wei, W. Keipper et al., High energy-storage density under low electric fields and improved optical transparency in novel sodium bismuth titanate-based lead-free ceramics. J. Eur. Ceram. Soc. **40**, 71–77 (2020). https://doi.org/10.1016/j.jeurceramsoc.2019.09.001

[S15] M. Chen, Y. Pu, L. Zhang, Novel NBT-based relaxor ferroelectric ceramics with excellent discharge performance and high-temperature stability. J. Mater. Sci. Mater. Electron. **32**, 23540–23553 (2021). https://doi.org/10.1007/s10854-021-06841-2

[S16] Z. Jiang, Z. Yang, Y. Yuan, B. Tang, S. Zhang, High energy storage properties and dielectric temperature stability of (1-x)($0.8Bi_{0.5}Na_{0.5}TiO_3$-$0.2Ba_{0.3}Sr_{0.7}$TiO3)-x$NaNbO_3$ lead-free ceramics. J. Alloys Compd. **851**, 156821 (2021). https://doi.org/10.1016/j.jallcom.2020.156821

[S17] L. Zhang, Z. Wang, Y. Li, P. Chen, J. Cai et al., Enhanced energy storage performance in Sn doped $Sr_{0.6}(Na_{0.5}Bi_{0.5})_{0.4}TiO_3$ lead-free relaxor ferroelectric ceramics. J. Eur. Ceram. Soc. **39**, 3057–3063 (2019). https://doi.org/10.1016/j.jeurceramsoc.2019.02.004

[S18] M.K. Bilal, J. Wang, R. Bashir, H. Liu, S.U. Asif et al., A novel relaxor (Bi, Na, Ba)(Ti, Zr)O_3 lead-free ceramic with high energy storage performance. J. Am. Ceram. Soc. **104**, 3982–3991 (2021). https://doi.org/10.1111/jace.17785

[S19] Z. Pan, D. Hu, Y. Zhang, J. Liu, B. Shen et al., Achieving high discharge energy density and efficiency with NBT-based ceramics for application in capacitors. J. Mater. Chem. C **7**, 4072–4078 (2019). https://doi.org/10.1039/c9tc00087a

[S20] X. Zhou, H. Qi, Z. Yan, G. Xue, H. Luo, D. Zhang, Large energy density with excellent stability in fine-grained (Bi0.5Na0.5)TiO3-based lead-free ceramics, Journal of the European Ceramic Society 39 (2019) 4053-4059, https://doi.org/10.1016/j.jeurceramsoc.2019.05.056

[S21] M. Wang, Q. Feng, Y. Wei, N. Luo, C. Yuan et al., Relaxor ferroelectric $Bi_{0.5}Na_{0.5}TiO_3$–$Sr_{0.7}Nd_{0.2}TiO_3$ ceramics with high energy storage density and excellent

stability under a low electric field. J. Phys. Chem. Solids **157**, 110209 (2021). https://doi.org/10.1016/j.jpcs.2021.110209

[S22] J. Xie, Z. Dai, X. Ding, X. Fan, W. Liu et al., Enhanced energy storage properties of $Sr(Sc_{0.5}Nb_{0.5})O_3$ modified $(Bi_{0.47}La_{0.03}Na_{0.5})_{0.94}Ba_{0.06}TiO3$ lead-free ceramics. J. Mater. Sci. **55**, 13578–13589 (2020). https://doi.org/10.1007/s10853-020-04978-9

[S23] X. Zhao, C. Li, J. Liu, Y. Ding, W. Bai et al., $(Bi_{0.5}Na_{0.5})TiO_3$-based relaxor ferroelectrics with simultaneous high energy storage properties and remarkable charge-discharge performances under low working electric fields for dielectric capacitor applications. Ceram. Int. **47**, 25800–25809 (2021). https://doi.org/10.1016/j.ceramint.2021.05.308

[S24] L. Zhang, X. Pu, M. Chen, S. Bai, Y. Pu, Influence of BaSnO 3 additive on the energy storage properties of $Na_{0.5}$ $Bi_{0.5}$ TiO 3-based relaxor ferroelectrics. J. Eur. Ceram. Soc. **38**, 2304–2311 (2018). https://doi.org/10.1016/j.jeurceramsoc.2017.11.053

[S25] Y. Pu, L. Zhang, Y. Cui, M. Chen, High energy storage density and optical transparency of microwave sintered homogeneous $(Na_{0.5}Bi_{0.5})_{(1-x)}Ba_xTi_{(1-y)}Sn_yO_3$ ceramics. ACS Sustainable Chem. Eng. **6**, 6102–6109 (2018). https://doi.org/10.1021/acssuschemeng.7b04754

[S26] G. Liu, J. Dong, L. Zhang, L. Yu, F. Wei et al., $Na_{0.25}Sr_{0.5}Bi_{0.25}TiO_3$ relaxor ferroelectric ceramic with greatly enhanced electric storage property by a B-site ion doping. Ceram. Int. **46**, 11680–11688 (2020). https://doi.org/10.1016/j.ceramint.2020.01.199

[S27] L. Zhang, Y. Pu, M. Chen, T. Wei, X. Peng, Novel $Na_{0.5}Bi_{0.5}TiO_3$ based, lead-free energy storage ceramics with high power and energy density and excellent high-temperature stability. Chem. Eng. J. **383**, 123154 (2020). https://doi.org/10.1016/j.cej.2019.123154

[S28] J. Li, F. Li, Z. Xu, S. Zhang, Antiferroelectrics: multilayer lead-free ceramic capacitors with ultrahigh energy density and efficiency. Adv. Mater. **30**, 1870240 (2018). https://doi.org/10.1002/adma.201870240

[S29] Y. Du, Y. Qi, L. Hu, H. Wan, H. Wang et al., $Na_{0.5}Bi_{0.5}TiO_3$-based ferroelectric relaxor with high thermal stability and anti-fatigue for charge-discharge properties. Ceram. Int. **47**, 15834–15842 (2021). https://doi.org/10.1016/j.ceramint.2021.02.157

[S30] T. Wang, J. Liu, L. Kong, H. Yang, F. Wang et al., Evolution of the structure, dielectric and ferroelectric properties of $Na_{0.5}Bi_{0.5}TiO_3$-added $BaTiO_3$–$Bi(Mg_{2/3}Nb_{1/3})O_3$ ceramics. Ceram. Int. **46**, 25392–25398 (2020). https://doi.org/10.1016/j.ceramint.2020.07.007

[S31] X. Zhao, W. Bai, Y. Ding, L. Wang, S. Wu et al., Tailoring high energy density with superior stability under low electric field in novel $(Bi_{0.5}Na_{0.5})TiO_3$-based relaxor ferroelectric ceramics. J. Eur. Ceram. Soc. **40**, 4475–4486 (2020). https://doi.org/10.1016/j.jeurceramsoc.2020.05.078

[S32] X. Fan, P. Li, J. Du, C. Chen, P. Fu et al., High-energy storage performance of (1–x)[0.935($Bi_{0.5}Na_{0.5}$)TiO3–0.065$BaTiO_3$]–xBa($Zr_{0.3}Ti_{0.7}$)O_3 ceramics with wide temperature range. J. Mater. Sci. Mater. Electron. **31**, 9974–9981 (2020). https://doi.org/10.1007/s10854-020-03602-5

[S33] F. Yang, Z. Pan, Z. Ling, D. Hu, J. Ding et al., Realizing high comprehensive energy storage performances of BNT-based ceramics for application in pulse power capacitors. J. Eur. Ceram. Soc. **41**, 2548–2558 (2021). https://doi.org/10.1016/j.jeurceramsoc.2020.11.049

[S34] Y. Yang, J. Xu, L. Yang, C. Zhou, H. Zhang et al., Highly enhanced discharged energy density and superior cyclic stability of $Bi_{0.5}Na_{0.5}TiO_3$-based ceramics by introducing $Sr_{0.7}Ca_{0.3}TiO_3$ component. Mater. Chem. Phys. **276**, 125402 (2022). https://doi.org/10.1016/j.matchemphys.2021.125402

[S35] Y. Huang, L. Zhang, R. Jing, Q. Hu, D.O. Alikin et al., Thermal stability of dielectric and energy storage performances of Ca-substituted BNTZ ferroelectric ceramics. Ceram. Int. **47**, 6298–6309 (2021). https://doi.org/10.1016/j.ceramint.2020.10.208

[S36] Z. He, S. Shi, Z. Pan, L. Tang, J. Zhao et al., Low electric field induced high energy storage capability of the free-lead relaxor ferroelectric 0.94$Bi_{0.5}Na_{0.5}TiO_3$-0.06$BaTiO_3$-based ceramics. Ceram. Int. **47**, 11611–11617 (2021). https://doi.org/10.1016/j.ceramint.2020.12.294

[S37] L. Zhang, Y. Pu, M. Chen, Ultra-high energy storage performance under low electric fields in $Na_{0.5}Bi_{0.5}TiO_3$-based relaxor ferroelectrics for pulse capacitor applications. Ceram. Int. **46**, 98–105 (2020). https://doi.org/10.1016/j.ceramint.2019.08.238

[S38] L. Zhang, Y. Pu, M. Chen, G. Liu, Antiferroelectric-like properties in MgO-modified 0.775$Na_{0.5}Bi_{0.5}TiO_3$-0.225$BaSnO_3$ ceramics for high power energy storage. J. Eur. Ceram. Soc. **38**, 5388–5395 (2018). https://doi.org/10.1016/j.jeurceramsoc.2018.08.010

[S39] F. Zhang, X. Qiao, Q. Zhou, Q. Shi, X. Chao et al., High energy and power density achieved in $Bi_{0.5}Na_{0.5}TiO_3$-based relaxor ferroelectric ceramics with excellent thermal stability. J. Alloys Compd. **875**, 160005 (2021). https://doi.org/10.1016/j.jallcom.2021.160005

[S40] Y. Wu, Y. Fan, N. Liu, P. Peng, M. Zhou et al., Enhanced energy storage properties in sodium bismuth titanate-based ceramics for dielectric capacitor applications. J. Mater. Chem. C **7**, 6222–6230 (2019). https://doi.org/10.1039/c9tc01239g

[S41] F. Zhang, X. Qiao, Q. Shi, X. Chao, Z. Yang et al., High energy storage density realized in $Bi_{0.5}Na_{0.5}TiO_3$-based relaxor ferroelectric ceramics at ultralow sintering temperature. J. Eur. Ceram. Soc. **41**, 368–375 (2021). https://doi.org/10.1016/j.jeurceramsoc.2020.07.067

[S42] C. Zhu, Z. Cai, B. Luo, L. Guo, L. Li et al., High temperature lead-free BNT-based ceramics with stable energy storage and dielectric properties. J. Mater. Chem. A **8**,

683–692 (2020). https://doi.org/10.1039/c9ta10347c

[S43] C. Cui, Y. Pu, Effect of Sn substitution on the energy storage properties of $0.45SrTiO_3$–$0.2Na_{0.5}Bi_{0.5}TiO3$–$0.35BaTiO_3$ ceramics. J. Mater. Sci. **53**, 9830–9841 (2018). https://doi.org/10.1007/s10853-018-2282-8

[S44] D. Hu, Z. Pan, Z. He, F. Yang, X. Zhang et al., Significantly improved recoverable energy density and ultrafast discharge rate of $Na_{0.5}Bi_{0.5}TiO_3$-based ceramics. Ceram. Int. **46**, 15364–15371 (2020). https://doi.org/10.1016/j.ceramint.2020.03.080

[S45] Y. Ding, J. Liu, C. Li, W. Bai, S. Wu et al., High capacitive performance at moderate operating field in $(Bi_{0.5}Na_{0.5})TiO_3$-based dielectric ceramics via synergistic effect of site engineering strategy. Chem. Eng. J. **426**, 130811 (2021). https://doi.org/10.1016/j.cej.2021.130811

[S46] L. Zhang, Y. Pu, M. Chen, Influence of BaZrO3 additive on the energy-storage properties of 0.775Na0.5Bi0.5TiO3-0.225BaSnO3 relaxor ferroelectrics, Journal of Alloys and Compounds 775 (2019) 342-347, https://doi.org/10.1016/j.jallcom.2018.10.025

[S47] X. Qiao, D. Wu, F. Zhang, B. Chen, X. Ren et al., $Bi_{0.5}Na_{0.5}TiO_3$-based relaxor ferroelectric ceramic with large energy density and high efficiency under a moderate electric field. J. Mater. Chem. C **7**, 10514–10520 (2019). https://doi.org/10.1039/c9tc03597d

[S48] K. Yao, C. Zhou, J. Wang, Y. Tan, Q. Li et al., $Bi_{0\cdot5}Na_{0\cdot5}TiO_3$–$Sr_{0.85}Bi_{0\cdot1}TiO_3$ ceramics with high energy storage properties and extremely fast discharge speed via regulating relaxation temperature. Ceram. Int. **47**, 11294–11303 (2021). https://doi.org/10.1016/j.ceramint.2020.12.255

[S49] Y. Lin, D. Li, M. Zhang, H. Yang, $(Na_{0.5}Bi_{0.5})_{0.7}Sr_{0.3}TiO_3$ modified by $Bi(Mg_{2/3}Nb_{1/3})O_3$ ceramics with high energy-storage properties and an ultrafast discharge rate. J. Mater. Chem. C **8**, 2258–2264 (2020). https://doi.org/10.1039/c9tc06218a

[S50] L. Zheng, P. Sun, P. Zheng, W. Bai, L. Li et al., Significantly tailored energy-storage performances in $Bi_{0.5}Na_{0.5}TiO_3$–$SrTiO_3$-based relaxor ferroelectric ceramics by introducing bismuth layer-structured relaxor $BaBi_2Nb_2O_9$ for capacitor application. J. Mater. Chem. C **9**, 5234–5243 (2021). https://doi.org/10.1039/d1tc00437a

[S51] H. Yang, J. Tian, Y. Lin, J. Ma, Realizing ultra-high energy storage density of lead-free $0.76Bi_{0.5}Na_{0.5}TiO_3$-$0.24SrTiO_3$-$Bi(Ni_{2/3}Nb_{1/3})O_3$ ceramics under low electric fields. Chem. Eng. J. **418**, 129337 (2021). https://doi.org/10.1016/j.cej.2021.129337

[S52] X. Qiao, A. Sheng, D. Wu, F. Zhang, B. Chen et al., A novel multifunctional ceramic with photoluminescence and outstanding energy storage properties. Chem. Eng. J. **408**, 127368 (2021). https://doi.org/10.1016/j.cej.2020.127368

[S53] W. Shi, Y. Yang, L. Zhang, R. Jing, Q. Hu et al., Enhanced energy storage performance of eco-friendly BNT-based relaxor ferroelectric ceramics via polarization

mismatch-reestablishment and viscous polymer process. Ceram. Int. **48**, 6512–6519 (2022). https://doi.org/10.1016/j.ceramint.2021.11.197

[S54] D. Li, Y. Lin, Q. Yuan, M. Zhang, L. Ma et al., A novel lead-free $Na_{0.5}Bi_{0.5}TiO_3$-based ceramic with superior comprehensive energy storage and discharge properties for dielectric capacitor applications. J. Materiomics **6**, 743–750 (2020). https://doi.org/10.1016/j.jmat.2020.06.005

[S55] X. Zhang, F. Yang, W. Miao, Z. Su, J. Zhao et al., Effective improved energy storage performances of $Na_{0.5}Bi_{0.5}TiO_3$-based relaxor ferroelectrics ceramics by A/B-sites co-doping. J. Alloys Compd. **883**, 160837 (2021). https://doi.org/10.1016/j.jallcom.2021.160837

[S56] Y. Huang, F. Li, H. Hao, F. Xia, H. Liu et al., $(Bi_{0.51}\ Na_{0.47})TiO_3$ based lead free ceramics with high energy density and efficiency. J. Materiomics **5**, 385–393 (2019). https://doi.org/10.1016/j.jmat.2019.03.006

[S57] Z. Ling, J. Ding, W. Miao, J. Liu, J. Zhao et al., MnO_2-modified lead-free NBT-based relaxor ferroelectric ceramics with improved energy storage performances. Ceram. Int. **47**, 22065–22072 (2021). https://doi.org/10.1016/j.ceramint.2021.04.227

[S58] [S58] C. Luo, Q. Feng, N. Luo, C. Yuan, C. Zhou, Y. Wei, T. Fujita, J. Xu, G. Chen, Effect of Ca2+/Hf4+ modification at A/B sites on energy-storage density of Bi0.47Na0.47Ba0.06TiO3 ceramics, Chemical Engineering Journal 420 (2021) 129861, https://doi.org/10.1016/j.cej.2021.129861

[S59] C. Cui, Y. Pu, Improvement of energy storage density with trace amounts of ZrO_2 additives fabricated by wet-chemical method. J. Alloys Compd. **747**, 495–504 (2018). https://doi.org/10.1016/j.jallcom.2018.03.058

[S60] Y. Wang, T. Wang, J. Wang, J. Liu, Z. Xing et al., A-site compositional modulation in Barium titanate based relaxor ceramics to achieve simultaneously high energy density and efficiency. J. Eur. Ceram. Soc. **41**, 6474–6481 (2021). https://doi.org/10.1016/j.jeurceramsoc.2021.05.052

[S61] T. Li, P. Chen, F. Li, C. Wang, Energy storage performance of $Na_{0.5}Bi_{0.5}TiO_3$-$SrTiO_3$ lead-free relaxors modified by $AgNb_{0.85}Ta_{0.15}O_3$. Chem. Eng. J. **406**, 127151 (2021). https://doi.org/10.1016/j.cej.2020.127151

[S62] X. Zhang, D. Hu, Z. Pan, X. Lv, Z. He et al., Enhancement of recoverable energy density and efficiency of lead-free relaxor-ferroelectric BNT-based ceramics. Chem. Eng. J. **406**, 126818 (2021). https://doi.org/10.1016/j.cej.2020.126818

[S63] [S63] W. Cao, T. Li, P. Chen, C. Wang, Outstanding energy storage performance of Na0.5Bi0.5TiO3-BaTiO3-(Sr0.85Bi0.1)(Mg1/3Nb2/3)O3 Lead-Free Ceramics. ACS Appl. Energy Mater. (2021), https://doi.org/10.1021/acsaem.1c01566

[S64] Z. Ma, Q. Su, J. Zhu, X. Meng, Y. Zhao et al., Optimization of energy-storage properties for lead-free relaxor-ferroelectric (1-x)$Na_0.5Bi_{0.5}TiO_{3\text{-}x}Sr_{0.7}Nd_{0.2}$TiO3 ceramics. J. Mater. Sci. **57**, 217–228 (2022). https://doi.org/10.1007/s10853-021-

06684-6

[S65] L. Yang, X. Kong, Z. Cheng, S. Zhang, Ultra-high energy storage performance with mitigated polarization saturation in lead-free relaxors. J. Mater. Chem. A **7**, 8573–8580 (2019). https://doi.org/10.1039/c9ta01165j

[S66] X. Qiao, F. Zhang, D. Wu, B. Chen, X. Zhao et al., Superior comprehensive energy storage properties in $Bi_{0.5}Na_{0.5}TiO_3$-based relaxor ferroelectric ceramics. Chem. Eng. J. **388**, 124158 (2020). https://doi.org/10.1016/j.cej.2020.124158

[S67] X. Guo, P. Shi, X. Lou, Q. Liu, H. Zuo, Superior energy storage properties in (1–x)(0.65$Bi_{0.5}Na_{0.5}TiO_3^{-}0.35Bi_{0.2}Sr_{0.7}$TiO3)-x$CaZrO_3$ ceramics with excellent temperature stability. J. Alloys Compd. **876**, 160101 (2021). https://doi.org/10.1016/j.jallcom.2021.160101

[S68] J. Zhu, Z. Ma, Q. Su, X. Meng, Y. Zhao et al., Enhanced energy-storage properties of lead-free $Bi_{0.5}Na_{0.5}TiO_3^{-}$ based relaxor ferroelectric ceramics by tuning sintering temperature. J. Mater. Sci. Mater. Electron. **32**, 26258–26267 (2021). https://doi.org/10.1007/s10854-021-06857-8

[S69] D. Hu, Z. Pan, X. Zhang, H. Ye, Z. He et al., Greatly enhanced discharge energy density and efficiency of novel relaxation ferroelectric BNT–BKT-based ceramics. J. Mater. Chem. C **8**, 591–601 (2020). https://doi.org/10.1039/c9tc05528b

[S70] P. Shi, T. Li, X. Lou, Z. Yu, X. Zhu, C. Zhou, Q. Liu, L. He, X. Zhang, S. Yang, Large electric-field-induced strain and energy storage properties in Bi0.5Na0.5TiO3-(0.5Ba0.7Ca0.3TiO3-0.5BaTi0.8Zr0.2O3) lead-free relaxor ferroelectric ceramics, Journal of Alloys and Compounds 860 (2021) 158369, https://doi.org/10.1016/j.jallcom.2020.158369

[S71] S. Bian, Z. Yue, Y. Shi, J. Zhang, W. Feng, Ultrahigh energy storage density and charge-discharge performance in novel sodium bismuth titanate-based ceramics. J. Am. Ceram. Soc. **104**, 936–947 (2021). https://doi.org/10.1111/jace.17486

[S72] P. Shi, X. Zhu, X. Lou, B. Yang, X. Guo et al., $Bi_{0.5}Na_{0.5}TiO_3$-based lead-free ceramics with superior energy storage properties at high temperatures. Compos. Part B Eng. **215**, 108815 (2021). https://doi.org/10.1016/j.compositesb.2021.108815

[S73] X. Zhou, H. Qi, Z. Yan, G. Xue, H. Luo et al., Superior thermal stability of high energy density and power density in domain-engineered $Bi_{0.5}Na_{0.5}TiO_3$–$NaTaO_3$ relaxor ferroelectrics. ACS Appl. Mater. Interfaces **11**, 43107–43115 (2019). https://doi.org/10.1021/acsami.9b13215

[S74] P. Shi, X. Zhu, X. Lou, B. Yang, Q. Liu et al., Tailoring ferroelectric polarization and relaxation of BNT-based lead-free relaxors for superior energy storage properties. Chem. Eng. J. **428**, 132612 (2022). https://doi.org/10.1016/j.cej.2021.132612

[S75] B. Guo, Y. Yan, M. Tang, Z. Wang, Y. Li et al., Energy storage performance of $Na_{0.5}Bi_{0.5}TiO_3$ based lead-free ferroelectric ceramics prepared via non-uniform phase structure modification and rolling process. Chem. Eng. J. **420**, 130475 (2021).

https://doi.org/10.1016/j.cej.2021.130475

[S76] D. Li, D. Zhou, W. Liu, P.-J. Wang, Y. Guo et al., Enhanced energy storage properties achieved in $Na_{0.5}Bi_{0.5}TiO_3$-based ceramics via composition design and domain engineering. Chem. Eng. J. **419**, 129601 (2021). https://doi.org/10.1016/j.cej.2021.129601

[S77] X. Li, Y. Cheng, F. Wang, Q. Xu, Y. Chen et al., Enhancement of energy storage and hardness of $(Na_{0.5}Bi_{0.5})_{0.7}Sr_{0.3}TiO_3$-based relaxor ferroelectrics via introducing $Ba(Mg_{1/3}Nb_{2/3})O_3$. Chem. Eng. J. **431**, 133441 (2022). https://doi.org/10.1016/j.cej.2021.133441

[S78] F. Yan, X. Zhou, X. He, H. Bai, S. Wu et al., Superior energy storage properties and excellent stability achieved in environment-friendly ferroelectrics via composition design strategy. Nano Energy **75**, 105012 (2020). https://doi.org/10.1016/j.nanoen.2020.105012

[S79] F. Yan, K. Huang, T. Jiang, X. Zhou, Y. Shi et al., Significantly enhanced energy storage density and efficiency of BNT-based perovskite ceramics via A-site defect engineering. Energy Storage Mater. **30**, 392–400 (2020). https://doi.org/10.1016/j.ensm.2020.05.026

[S80] F. Yan, X. He, H. Bai, B. Shen, J. Zhai, Excellent energy storage properties and superior stability achieved in lead-free ceramics *via* a spatial sandwich structure design strategy. J. Mater. Chem. A **9**, 15827–15835 (2021). https://doi.org/10.1039/d1ta02853g

[S81] C. Zhang, W. Xiao, F. Zeng, D. Su, K. Du et al., Superior energy-storage performance in $0.85Bi_{0.5}Na_{0.5}TiO_3$–$0.15NaNbO_3$ lead-free ferroelectric ceramics *via* composition and microstructure engineering. J. Mater. Chem. A **9**, 10088–10094 (2021). https://doi.org/10.1039/d1ta01442k

[S82] F. Yan, H. Bai, X. Zhou, G. Ge, G. Li et al., Realizing superior energy storage properties in lead-free ceramics *via* a macro-structure design strategy. J. Mater. Chem. A **8**, 11656–11664 (2020). https://doi.org/10.1039/d0ta03526b

[S83] A.C.L.R.B.V. Dreele, Los Alamos National Laboratory Report, 1987, pp. No. LAUR-86–748.

[S84] X. Qiao, D. Wu, F. Zhang, B. Chen, X. Ren et al., $Bi_{0.5}Na_{0.5}TiO_3$-based relaxor ferroelectric ceramic with large energy density and high efficiency under a moderate electric field. J. Mater. Chem. C **7**, 10514–10520 (2019). https://doi.org/10.1039/c9tc03597d

[S85] L. Yang, X. Kong, Z. Cheng, S. Zhang, Ultra-high energy storage performance with mitigated polarization saturation in lead-free relaxors. J. Mater. Chem. A **7**, 8573–8580 (2019). https://doi.org/10.1039/c9ta01165j

[S86] H. Qi, R. Zuo, Linear-like lead-free relaxor antiferroelectric $(Bi_{0.5}Na_{0.5})TiO_3$–$NaNbO_3$ with giant energy-storage density/efficiency and super stability against temperature

and frequency. J. Mater. Chem. A **7**, 3971–3978 (2019). https://doi.org/10.1039/c8ta12232f

[S87] H. Yang, F. Yan, Y. Lin, T. Wang, Novel strontium titanate-based lead-free ceramics for high-energy storage applications. ACS Sustainable Chem. Eng. **5**, 10215–10222 (2017). https://doi.org/10.1021/acssuschemeng.7b02203

[S88] G. Liu, L. Hu, Y. Wang, Z. Wang, L. Yu et al., Investigation of electrical and electric energy storage properties of La-doped $Na_{0.3}$ $Sr_{0.4}Bi_{0.3}TiO_3$ based Pb-free ceramics. Ceram. Int. **46**, 19375–19384 (2020). https://doi.org/10.1016/j.ceramint.2020.04.280

[S89] L. Yu, J. Dong, M. Tang, Y. Liu, F. Wu et al., Enhanced electrical energy storage performance of Pb-free A-site La^{3+}-doped $0.85Na_{0.5}Bi_{0.5}TiO_3$-$0.15CaTiO_3$ ceramics. Ceram. Int. **46**, 28173–28182 (2020). https://doi.org/10.1016/j.ceramint.2020.07.316

[S90] T. Li, P. Chen, F. Li, C. Wang, Energy storage performance of $Na_{0.5}Bi_{0.5}TiO_3$-$SrTiO_3$ lead-free relaxors modified by $AgNb_{0.85}Ta_{0.15}O_3$. Chem. Eng. J. **406**, 127151 (2021). https://doi.org/10.1016/j.cej.2020.127151

[S91] Z. Jiang, H. Yang, L. Cao, Z. Yang, Y. Yuan et al., Enhanced breakdown strength and energy storage density of lead-free $Bi_{0.5}Na_{0.5}TiO_3$-based ceramic by reducing the oxygen vacancy concentration. Chem. Eng. J. **414**, 128921 (2021). https://doi.org/10.1016/j.cej.2021.128921

[S92] H. Ye, F. Yang, Z. Pan, D. Hu, X. Lv et al., Significantly improvement of comprehensive energy storage performances with lead-free relaxor ferroelectric ceramics for high-temperature capacitors applications. Acta Mater. **203**, 116484 (2021). https://doi.org/10.1016/j.actamat.2020.116484

[S93] D. Hu, Z. Pan, X. Zhang, H. Ye, Z. He et al., Greatly enhanced discharge energy density and efficiency of novel relaxation ferroelectric BNT–BKT-based ceramics. J. Mater. Chem. C **8**, 591–601 (2020). https://doi.org/10.1039/c9tc05528b

[S94] Y. Lin, D. Li, M. Zhang, H. Yang, $(Na_{0.5}Bi_{0.5})_{0.7}Sr_{0.3}TiO_3$ modified by $Bi(Mg_{2/3}Nb_{1/3})O_3$ ceramics with high energy-storage properties and an ultrafast discharge rate. J. Mater. Chem. C **8**, 2258–2264 (2020). https://doi.org/10.1039/c9tc06218a

[S95] R. Shi, Y. Pu, W. Wang, X. Guo, J. Li et al., A novel lead-free $NaNbO_3$–$Bi(Zn_{0.5}Ti_{0.5})O_3$ ceramics system for energy storage application with excellent stability. J. Alloys Compd. **815**, 152356 (2020). https://doi.org/10.1016/j.jallcom.2019.152356

[S96] J. Ye, G. Wang, M. Zhou, N. Liu, X. Chen et al., Excellent comprehensive energy storage properties of novel lead-free $NaNbO_3$-based ceramics for dielectric capacitor applications. J. Mater. Chem. C **7**, 5639–5645 (2019). https://doi.org/10.1039/c9tc01414d

[S97] M. Zhou, R. Liang, Z. Zhou, S. Yan, X. Dong, Novel sodium niobate-based lead-free ceramics as new environment-friendly energy storage materials with high energy

density, high power density, and excellent stability. ACS Sustainable Chem. Eng. **6**, 12755–12765 (2018). https://doi.org/10.1021/acssuschemeng.8b01926

[S98] C. Sun, X. Chen, J. Shi, F. Pang, X. Dong et al., Simultaneously with large energy density and high efficiency achieved in $NaNbO_3$-based relaxor ferroelectric ceramics. J. Eur. Ceram. Soc. **41**, 1891–1903 (2021). https://doi.org/10.1016/j.jeurceramsoc.2020.10.049

[S99] M. Zhou, R. Liang, Z. Zhou, X. Dong, Superior energy storage properties and excellent stability of novel $NaNbO_3$-based lead-free ceramics with A-site vacancy obtained *via* a Bi_2O_3 substitution strategy. J. Mater. Chem. A **6**, 17896–17904 (2018). https://doi.org/10.1039/c8ta07303a

[S100] L. Yang, X. Kong, Z. Cheng, S. Zhang, Enhanced energy density and electric cycling reliability via MnO_2 modification in sodium niobate-based relaxor dielectric capacitors. J. Mater. Res. **36**, 1214–1222 (2021). https://doi.org/10.1557/s43578-020-00085-2

[S101] L. Yang, X. Kong, Z. Cheng, S. Zhang, Enhanced energy storage performance of sodium niobate-based relaxor dielectrics by a ramp-to-spike sintering profile. ACS Appl. Mater. Interfaces **12**, 32834–32841 (2020). https://doi.org/10.1021/acsami.0c08737

[S102] X. Dong, X. Li, X. Chen, H. Chen, C. Sun et al., High energy storage density and power density achieved simultaneously in $NaNbO_3$-based lead-free ceramics via antiferroelectricity enhancement. J. Materiomics **7**, 629–639 (2021). https://doi.org/10.1016/j.jmat.2020.11.016

[S103] J. Shi, X. Chen, X. Li, J. Sun, C. Sun et al., Realizing ultrahigh recoverable energy density and superior charge–discharge performance in $NaNbO_3$-based lead-free ceramics *via* a local random field strategy. J. Mater. Chem. C **8**, 3784–3794 (2020). https://doi.org/10.1039/c9tc06711f

[S104] A.Tian, R. Zuo, H. Qi, M. Shi, Large energy-storage density in transition-metal oxide modified $NaNbO_3$–$Bi(Mg_{0.5}Ti_{0.5})O_3$lead-free ceramics through regulating the antiferroelectric phase structure. J. Mater. Chem. A **8**, 8352–8359 (2020). https://doi.org/10.1039/d0ta02285c

[S105] J. Chen, H. Qi, R. Zuo, Realizing stable relaxor antiferroelectric and superior energy storage properties in $(Na_{1-x/2}La_{x/2})(Nb_{1-x}Ti_x)O_3$ lead-free ceramics through A/B-site complex substitution. ACS Appl. Mater. Interfaces **12**, 32871–32879 (2020). https://doi.org/10.1021/acsami.0c09876

[S106] H. Chen, J. Shi, X. Chen, C. Sun, F. Pang et al., Excellent energy storage properties and stability of $NaNbO_3$–$Bi(Mg_{0.5}Ta_{0.5})O_3$ ceramics by introducing $(Bi_{0.5}Na_{0.5})_{0.7}Sr_{0.3}TiO_3$. J. Mater. Chem. A **9**, 4789–4799 (2021). https://doi.org/10.1039/d0ta11022a

[S107] Z. Chen, S. Mao, L. Ma, G. Luo, Q. Feng et al., Phase engineering in $NaNbO_3$ antiferroelectrics for high energy storage density. J. Materiomics **8**, 753–762 (2022). https://doi.org/10.1016/j.jmat.2022.03.004

[S108] J. Liu, P. Li, C. Li, W. Bai, S. Wu et al., Synergy of a stabilized antiferroelectric phase and domain engineering boosting the energy storage performance of $NaNbO_3$-based relaxor antiferroelectric ceramics. ACS Appl. Mater. Interfaces **14**, 17662–17673 (2022). https://doi.org/10.1021/acsami.2c01507

[S109] A. Xie, J. Fu, R. Zuo, Achieving stable relaxor antiferroelectric P phase in $NaNbO_3$-based lead-free ceramics for energy-storage applications. J. Materiomics **8**, 618–626 (2022). https://doi.org/10.1016/j.jmat.2021.11.012

[S110] T. Wang, L. Jin, C. Li, Q. Hu, X. Wei, Relaxor ferroelectric $BaTiO_3$–$Bi(Mg_{2/3}Nb_{1/3})O_3$ ceramics for energy storage application. J. Am. Ceram. Soc. **98**, 559–566 (2015). https://doi.org/10.1111/jace.13325

[S111] H. Yang, F. Yan, G. Zhang, Y. Lin, F. Wang, Dielectric behavior and impedance spectroscopy of lead-free $Ba_{0.85}Ca_{0.15}Zr_{0.1}Ti_{0.9}O_3$ ceramics with B_2O_3-Al_2O_3-SiO_2 glass-ceramics addition for enhanced energy storage. J. Alloys Compd. **720**, 116–125 (2017). https://doi.org/10.1016/j.jallcom.2017.05.158

[S112] F. Li, M. Zhou, J. Zhai, B. Shen, H. Zeng, Novel Barium titanate based ferroelectric relaxor ceramics with superior charge-discharge performance. J. Eur. Ceram. Soc. **38**, 4646–4652 (2018). https://doi.org/10.1016/j.jeurceramsoc.2018.06.038

[S113] L. Zhang, L.-X. Pang, W.-B. Li, D. Zhou, Extreme high energy storage efficiency in perovskite structured (1-x)$(Ba_{0.8}Sr_{0.2})TiO_{3\text{-}x}Bi(Zn_{2/3}Nb_{1/3})O_3$ (0.04≤x≤0.16) ceramics. J. Eur. Ceram. Soc. **40**, 3343–3347 (2020). https://doi.org/10.1016/j.jeurceramsoc.2020.03.015

[S114] W.-B. Li, D. Zhou, L.-X. Pang, Enhanced energy storage density by inducing defect dipoles in lead free relaxor ferroelectric $BaTiO_3$-based ceramics. Appl. Phys. Lett. **110,** (2017). https://doi.org/10.1063/1.4979467

[S115] H. Yang, F. Yan, Y. Lin, T. Wang, F. Wang et al., Lead-free BaTiO 3-Bi 0.5 $Na_{0.5}$ TiO 3-Na 0.73 $Bi_{0.09}$ NbO 3 relaxor ferroelectric ceramics for high energy storage. J. Eur. Ceram. Soc. **37**, 3303–3311 (2017). https://doi.org/10.1016/j.jeurceramsoc.2017.03.071

[S116] J. Wan, Y. Pu, C. Hui, C. Cui, Y. Guo, Synthesis and characterizations of $NaNbO_3$ modified $0.92BaTiO_3$–$0.08K_{0.5}Bi_{0.5}$TiO3 ceramics for energy storage applications. J. Mater. Sci. Mater. Electron. **29**, 5158–5162 (2018). https://doi.org/10.1007/s10854-017-8480-3

[S117] W.-B. Li, D. Zhou, L.-X. Pang, R. Xu, H.-H. Guo, Novel Barium titanate based capacitors with high energy density and fast discharge performance. J. Mater. Chem. A **5**, 19607–19612 (2017). https://doi.org/10.1039/c7ta05392d

[S118] W.-B. Li, D. Zhou, R. Xu, L.-X. Pang, I.M. Reaney, $BaTiO_3$–$Bi(Li_{0.5}Ta_{0.5})O_3$, lead-free ceramics, and multilayers with high energy storage density and efficiency. ACS Appl. Energy Mater. **1**, 5016–5023 (2018). https://doi.org/10.1021/acsaem.8b01001

[S119] Q. Yuan, F. Yao, Y. Wang, R. Ma, H. Wang, Relaxor ferroelectric $0.9BaTiO_3$–$0.1Bi(Zn_{0.5}Zr_{0.5})O_3$ ceramic capacitors with high energy density and temperature stable energy storage properties. J. Mater. Chem. C **5**, 9552–9558 (2017). https://doi.org/10.1039/c7tc02478a

[S120] H. Yang, Z. Lu, L. Li, W. Bao, H. Ji et al., Novel $BaTiO_3$-based, Ag/Pd-compatible lead-free relaxors with superior energy storage performance. ACS Appl. Mater. Interfaces **12**, 43942–43949 (2020). https://doi.org/10.1021/acsami.0c13057

[S121] Q. Hu, Y. Tian, Q. Zhu, J. Bian, L. Jin et al., Achieve ultrahigh energy storage performance in $BaTiO_3$–$Bi(Mg_{1/2}Ti_{1/2})O_3$ relaxor ferroelectric ceramics via nano-scale polarization mismatch and reconstruction. Nano Energy **67**, 104264 (2020). https://doi.org/10.1016/j.nanoen.2019.104264

[S122] G. Liu, Y. Li, B. Guo, M. Tang, Q. Li et al., Ultrahigh dielectric breakdown strength and excellent energy storage performance in lead-free Barium titanate-based relaxor ferroelectric ceramics via a combined strategy of composition modification, viscous polymer processing, and liquid-phase sintering. Chem. Eng. J. **398**, 125625 (2020). https://doi.org/10.1016/j.cej.2020.125625

[S123] Z. Dai, J. Xie, W. Liu, X. Wang, L. Zhang et al., Effective strategy to achieve excellent energy storage properties in lead-free $BaTiO_3$-based bulk ceramics. ACS Appl. Mater. Interfaces **12**, 30289–30296 (2020). https://doi.org/10.1021/acsami.0c02832

[S124] L. Chen, F. Li, B. Gao, C. Zhou, J. Wu et al., Excellent energy storage and mechanical performance in hetero-structure $BaTiO_3$-based relaxors. Chem. Eng. J. **452**, 139222 (2023). https://doi.org/10.1016/j.cej.2022.139222

[S125] T. Wang, L. Jin, Y. Tian, L. Shu, Q. Hu et al., Microstructure and ferroelectric properties of Nb_2O_5-modified $BiFeO_3$-$BaTiO_3$ lead-free ceramics for energy storage. Mater. Lett. **137**, 79–81 (2014). https://doi.org/10.1016/j.matlet.2014.08.133

[S126] D. Wang, Z. Fan, D. Zhou, A. Khesro, S. Murakami et al., Bismuth ferrite-based lead-free ceramics and multilayers with high recoverable energy density. J. Mater. Chem. A **6**, 4133–4144 (2018). https://doi.org/10.1039/c7ta09857j

[S127] G. Wang, Z. Lu, H. Yang, H. Ji, A. Mostaed et al., Fatigue resistant lead-free multilayer ceramic capacitors with ultrahigh energy density. J. Mater. Chem. A **8**, 11414–11423 (2020). https://doi.org/10.1039/d0ta00216j

[S128] H. Yang, H. Qi, R. Zuo, Enhanced breakdown strength and energy storage density in a new $BiFeO_3$-based ternary lead-free relaxor ferroelectric ceramic. J. Eur. Ceram. Soc. **39**, 2673–2679 (2019). https://doi.org/10.1016/j.jeurceramsoc.2019.03.001

[S129] Q. Li, S. Ji, D. Wang, J. Zhu, L. Li et al., Simultaneously enhanced energy storage

density and efficiency in novel $BiFeO_3$-based lead-free ceramic capacitors. J. Eur. Ceram. Soc. **41**, 387–393 (2021). https://doi.org/10.1016/j.jeurceramsoc.2020.08.032

[S130] N. Liu, R. Liang, Z. Zhou, X. Dong, Designing lead-free bismuth ferrite-based ceramics learning from relaxor ferroelectric behavior for simultaneous high energy density and efficiency under low electric field. J. Mater. Chem. C **6**, 10211–10217 (2018). https://doi.org/10.1039/c8tc03855d

[S131] G. Wang, J. Li, X. Zhang, Z. Fan, F. Yang et al., Ultrahigh energy storage density lead-free multilayers by controlled electrical homogeneity. Energy Environ. Sci. **12**, 582–588 (2019). https://doi.org/10.1039/c8ee03287d

[S132] F. Yan, H. Bai, G. Ge, J. Lin, C. Shi et al., Composition and structure optimized $BiFeO_3$-$SrTiO_3$ lead-free ceramics with ultrahigh energy storage performance. Small **18**, 2106515 (2022). https://doi.org/10.1002/smll.202106515

[S133] M. Wang, A. Xie, J. Fu, R. Zuo, Energy storage properties under moderate electric fields in $BiFeO_3$-based lead-free relaxor ferroelectric ceramics. Chem. Eng. J. **440**, 135789 (2022). https://doi.org/10.1016/j.cej.2022.135789

[S134] A. Khesro, F. Ahmad Khan, R. Muhammad, A. Ali, M. Khan et al., Energy storage performance of Nd^{3+}-doped $BiFeO_3$–$BaTiO_3$-based lead-free ceramics. Ceram. Int. **48**, 29938–29943 (2022). https://doi.org/10.1016/j.ceramint.2022.06.260

[S135] Y.H. Huang, Y.J. Wu, W.J. Qiu, J. Li, X.M. Chen, Enhanced energy storage density of $Ba_{0.4}Sr_{0.6}$TiO3–MgO composite prepared by spark plasma sintering. J. Eur. Ceram. Soc. **35**, 1469–1476 (2015). https://doi.org/10.1016/j.jeurceramsoc.2014.11.022

[S136] Y.H. Huang, Y.J. Wu, B. Liu, T.N. Yang, J.J. Wang et al., From core–shell $Ba_{0.4}Sr_{0.6}TiO_3$@SiO_2 particles to dense ceramics with high energy storage performance by spark plasma sintering. J. Mater. Chem. A **6**, 4477–4484 (2018). https://doi.org/10.1039/c7ta10821d

[S137] C. Cui, Y. Pu, Z. Gao, J. Wan, Y. Guo et al., Structure, dielectric and relaxor properties in lead-free ST-NBT ceramics for high energy storage applications. J. Alloys Compd. **711**, 319–326 (2017). https://doi.org/10.1016/j.jallcom.2017.04.023

[S138] C. Cui, Y. Pu, R. Shi, High-energy storage performance in lead-free (0.8-x)$SrTiO_3$-0.2$Na_{0.5}Bi_{0.5}TiO_{3\text{-}x}BaTiO_3$ relaxor ferroelectric ceramics. J. Alloys Compd. **740**, 1180–1187 (2018). https://doi.org/10.1016/j.jallcom.2018.01.106

[S139] Z. Yao, Q. Luo, G. Zhang, H. Hao, M. Cao et al., Improved energy-storage performance and breakdown enhancement mechanism of Mg-doped $SrTiO_3$ bulk ceramics for high energy density capacitor applications. J. Mater. Sci. Mater. Electron. **28**, 11491–11499 (2017). https://doi.org/10.1007/s10854-017-6945-z

[S140] W.-B. Li, D. Zhou, L.-X. Pang, Structure and energy storage properties of Mn-doped (Ba, Sr)TiO3–MgO composite ceramics. J. Mater. Sci. Mater. Electron. **28**, 8749–8754 (2017). https://doi.org/10.1007/s10854-017-6600-8

[S141] J. Qi, M. Cao, J.P. Heath, J.S. Dean, H. Hao et al., Improved breakdown strength and energy storage density of a Ce doped strontium titanate core by silica shell coating. J. Mater. Chem. C **6**, 9130–9139 (2018). https://doi.org/10.1039/c8tc03181a

[S142] W. Pan, M. Cao, A. Jan, H. Hao, Z. Yao et al., High breakdown strength and energy storage performance in (Nb, Zn) modified $SrTiO_3$ ceramics *via* synergy manipulation. J. Mater. Chem. C **8**, 2019–2027 (2020). https://doi.org/10.1039/c9tc06256d

[S143] H. Yang, F. Yan, Y. Lin, T. Wang, Enhanced recoverable energy storage density and high efficiency of $SrTiO_3$-based lead-free ceramics. Appl. Phys. Lett. **111,** 253903 (2017). https://doi.org/10.1063/1.5000980

[S144] C. Cui, Y. Pu, Improvement of energy storage density with trace amounts of ZrO_2 additives fabricated by wet-chemical method. J. Alloys Compd. **747**, 495–504 (2018). https://doi.org/10.1016/j.jallcom.2018.03.058

[S145] X. Kong, L. Yang, Z. Cheng, S. Zhang, Bi-modified $SrTiO_3$-based ceramics for high-temperature energy storage applications. J. Am. Ceram. Soc. **103**, 1722–1731 (2020). https://doi.org/10.1111/jace.16844

[S146] G. Dong, S. Ma, J. Du, J. Cui, Dielectric properties and energy storage density in ZnO-doped $Ba_{0.3}Sr_{0.7}TiO_3$ ceramics. Ceram. Int. **35**, 2069–2075 (2009). https://doi.org/10.1016/j.ceramint.2008.11.003

[S147] J. Wang, C. Xu, B. Shen, J. Zhai, Enhancing energy storage density of (Ba, Sr)TiO3 ceramic particles by coating with Al_2O_3 and SiO_2. J. Mater. Sci. Mater. Electron. **24**, 3309–3314 (2013). https://doi.org/10.1007/s10854-013-1248-5

[S148] H. Tao, J. Wu, Optimization of energy storage density in relaxor (K, Na, Bi)NbO_3 ceramics. J. Mater. Sci. Mater. Electron. **28**, 16199–16204 (2017). https://doi.org/10.1007/s10854-017-7521-2

[S149] X. Qiao, X. Zhang, D. Wu, X. Chao, Z. Yang, Influence of Bi nonstoichiometry on the energy storage properties of 0.93KNN–0.07Bi_xMN relaxor ferroelectrics. J. Adv. Dielect. **8**, 1830006 (2018). https://doi.org/10.1142/s2010135x18300062

[S150] Z. Yang, F. Gao, H. Du, L. Jin, L. Yan et al., Grain size engineered lead-free ceramics with both large energy storage density and ultrahigh mechanical properties. Nano Energy **58**, 768–777 (2019). https://doi.org/10.1016/j.nanoen.2019.02.003

[S151] B. Qu, H. Du, Z. Yang, Q. Liu, T. Liu, Enhanced dielectric breakdown strength and energy storage density in lead-free relaxor ferroelectric ceramics prepared using transition liquid phase sintering. RSC Adv. **6**, 34381–34389 (2016). https://doi.org/10.1039/c6ra01919f

[S152] Y. Li, Y. Zhen, W. Wang, Z. Fang, Z. Jia et al., Enhanced energy storage density and discharge efficiency in potassium sodium niobite-based ceramics prepared using a new scheme. J. Eur. Ceram. Soc. **40**, 2357–2365 (2020). https://doi.org/10.1016/j.jeurceramsoc.2020.01.050

[S153] M. Zhang, H. Yang, D. Li, L. Ma, Y. Lin, Giant energy storage efficiency and high recoverable energy storage density achieved in $K_{0.5}Na_{0.5}NbO_3$-$Bi(Zn_{0.5}Zr_{0.5})O_3$ ceramics. J. Mater. Chem. C **8**, 8777–8785 (2020). https://doi.org/10.1039/d0tc01711f

[S154] B. Qu, H. Du, Z. Yang, Q. Liu, Large recoverable energy storage density and low sintering temperature in potassium-sodium niobate-based ceramics for multilayer pulsed power capacitors. J. Am. Ceram. Soc. **100**, 1517–1526 (2017). https://doi.org/10.1111/jace.14728

[S155] Z. Yang, H. Du, S. Qu, Y. Hou, H. Ma et al., Significantly enhanced recoverable energy storage density in potassium–sodium niobate-based lead free ceramics. J. Mater. Chem. A **4**, 13778–13785 (2016). https://doi.org/10.1039/c6ta04107h

[S156] T. Shao, H. Du, H. Ma, S. Qu, J. Wang et al., Potassium–sodium niobate based lead-free ceramics: novel electrical energy storage materials. J. Mater. Chem. A **5**, 554–563 (2017). https://doi.org/10.1039/c6ta07803f

[S157] Z. Dai, D. Li, Z. Zhou, S. Zhou, W. Liu et al., A strategy for high performance of energy storage and transparency in KNN-based ferroelectric ceramics. Chem. Eng. J. **427**, 131959 (2022). https://doi.org/10.1016/j.cej.2021.131959

[S158] S. Li, T. Hu, H. Nie, Z. Fu, C. Xu et al., Giant energy density and high efficiency achieved in silver niobate-based lead-free antiferroelectric ceramic capacitors *via* domain engineering. Energy Storage Mater. **34**, 417–426 (2021). https://doi.org/10.1016/j.ensm.2020.09.021

[S159] Z. Lu, W. Bao, G. Wang, S.-K. Sun, L. Li et al., Mechanism of enhanced energy storage density in $AgNbO_3$-based lead-free antiferroelectrics. Nano Energy **79**, 105423 (2021). https://doi.org/10.1016/j.nanoen.2020.105423

[S160] N. Luo, K. Han, M.J. Cabral, X. Liao, S. Zhang et al., Constructing phase boundary in $AgNbO_3$ antiferroelectrics: pathway simultaneously achieving high energy density and efficiency. Nat. Commun. **11**, 4824 (2020). https://doi.org/10.1038/s41467-020-18665-5

[S161] N. Luo, K. Han, F. Zhuo, C. Xu, G. Zhang et al., Correction: Aliovalent A-site engineered $AgNbO_3$ lead-free antiferroelectric ceramics toward superior energy storage density. J. Mater. Chem. A **7**, 15450 (2019). https://doi.org/10.1039/c9ta02053e

[S162] K. Han, N. Luo, S. Mao, F. Zhuo, L. Liu et al., Ultrahigh energy-storage density in A-/B-site Co-doped $AgNbO_3$ lead-free antiferroelectric ceramics: insight into the origin of antiferroelectricity. J. Mater. Chem. A **7**, 26293–26301 (2019). https://doi.org/10.1039/c9ta06457e

[S163] J. Gao, Q. Liu, J. Dong, X. Wang, S. Zhang et al., Local structure heterogeneity in Sm-doped $AgNbO_3$ for improved energy-storage performance. ACS Appl. Mater. Interfaces **12**, 6097–6104 (2020). https://doi.org/10.1021/acsami.9b20803

[S164] W. Chao, T. Yang, Y. Li, Z. Liu, Enhanced energy storage density in Ca and Ta Co-

doped $AgNbO_3$ antiferroelectric ceramics. J. Am. Ceram. Soc. **103**, 7283–7290 (2020). https://doi.org/10.1111/jace.17415

[S165] C. Xu, Z. Fu, Z. Liu, L. Wang, S. Yan et al., La/Mn codoped $AgNbO_3$ lead-free antiferroelectric ceramics with large energy density and power density. ACS Sustainable Chem. Eng. **6**, 16151–16159 (2018). https://doi.org/10.1021/acssuschemeng.8b02821

[S166] K. Han, N. Luo, S. Mao, F. Zhuo, X. Chen et al., Realizing high low-electric-field energy storage performance in $AgNbO_3$ ceramics by introducing relaxor behaviour. J. Materiomics **5**, 597–605 (2019). https://doi.org/10.1016/j.jmat.2019.07.006

[S167] A. Verma, A.K. Yadav, S. Kumar, V. Srihari, R. Jangir et al., Structural, thermally stable dielectric, and energy storage properties of lead-free $(1-x)(Na_{0.50}Bi_{0.50})TiO3-xKSbO3$ ceramics. J. Mater. Sci. Mater. Electron. **30**, 15005–15017 (2019). https://doi.org/10.1007/s10854-019-01873-1

[S168] Y. Tian, L. Jin, H. Zhang, Z. Xu, X. Wei et al., High energy density in silver niobate ceramics. J. Mater. Chem. A **4**, 17279–17287 (2016). https://doi.org/10.1039/c6ta06353e

[S169] D. Feng, H. Du, H. Ran, T. Lu, S. Xia et al., Antiferroelectric stability and energy storage properties of Co-doped $AgNbO_3$ ceramics. J. Solid State Chem. **310**, 123081 (2022). https://doi.org/10.1016/j.jssc.2022.123081

[S170] X. Fan, J. Wang, H. Yuan, L. Chen, L. Zhao et al., Synergic enhancement of energy storage density and efficiency in MnO_2-doped $AgNbO_3$@SiO_2 ceramics via A/B-site substitutions. ACS Appl. Mater. Interfaces **14**, 7052–7062 (2022). https://doi.org/10.1021/acsami.1c25234

[S171] A.K. Yadav, H. Fan, B. Yan, C. Wang, J. Ma et al., Enhanced storage energy density and fatigue free properties for $0.94Bi_{0.50}(Na_{0.78}K_{0.22})_{0.50}Ti_{1-x}(Al_{0.50}Nb_{0.50})_xO_3$-$0.06BaZrO_3$ ceramics. Ceram. Int. **46**, 17044–17052 (2020). https://doi.org/10.1016/j.ceramint.2020.03.292
